# Anatomy of adult *Megaphragma* (Hymenoptera: Trichogrammatidae), one of the smallest insects, and new insight into insect miniaturization

**DOI:** 10.1371/journal.pone.0175566

**Published:** 2017-05-03

**Authors:** Alexey A. Polilov

**Affiliations:** Department of Entomology, Faculty of Biology, Lomonosov Moscow State University, Moscow, Russia; Smithsonian National Museum of Natural History, UNITED STATES

## Abstract

The body size, especially in cases of extreme reduction, is an important characteristic that strongly determines the morphology, physiology, and biology of animals. Miniaturization is a widespread trend in animal evolution and one of the principal directions of evolution in insects. Miniaturization-related features of insect morphology have been subject to intensive studies during the last few years, but the structure of the smallest insects remains insufficiently known. It is especially important to study hymenopterans of the genus *Megaphragma*, which include the smallest flying insects and a species in which an almost anucleate nervous system was recently discovered. This article is the first detailed study of the external and internal morphology of adults of *Megaphragma mymaripenne* and *M*. *amalphitanum* using histological methods, 3D computer modeling and other techniques. It is shown that in spite of the extremely small size the organization of *Megaphragma* retains a considerkable level of structural complexity. On the other hand, miniaturization leads to re-organizations of several organ systems. Unique structural features related to miniaturization have been found in both species: lysis of cell bodies and nuclei of neurons at late stages of pupal development, absence of the heart, and considerable reductions in the set of muscles. Comparative analysis of structure in the smallest insects representing different taxa has revealed common features of the evolutionary process of miniaturization in insects.

## Introduction

Trichogrammatids of the genus *Megaphragma* include some of the smallest insects and some of the smallest metazoans. Most representatives of this genus have a body length of less than 300 μm and are comparable in size to some unicellular organisms [1]. A unique almost anucleate nervous system was recently described in *M*. *mymaripenne* [2]. Therefore, the study of these extremely miniaturized insects is of considerable interest in the context of miniaturization in insects and miniaturization of the nervous system in animals in general. However, the available data on the external morphology of representatives of this genus are limited only to brief diagnoses of species and descriptions of particular elements of morphology [3, 4, 5, 6]. Their internal structure has remained unstudied.

Since some trichogrammatids are widely used in biological pest control, their biology and taxonomy have been studied intensely [7]. Fewer studies treat the morphology of Trichogrammatidae. The external morphology of the adults of many genera has been described in some detail [5, 8, 9]. Only one detailed study on the species *Trichogramma evanescens* treats the internal morphology of adult trichogrammatids [10]. Other available publications on the structure of adult trichogrammatids include studies on specific aspects of the anatomy of different *Trichogramma* species [11, 12, 13] and *Prestwichia aquatica* [14], descriptions of the structure of the cerebrum in *Megaphragma mymaripenne* [2] and *Trichogramma evanescens* [15], descriptions of the eye structure in *Trichogramma* [16], and *Megaphragma* [17], and ultrastructure of the spermatozoa in *Trichogramma* [18, 19].

The main purpose of this study was to describe the external and internal morphology and to analyze miniaturization-related features in *Megaphragma*.

## Materials and methods

### Materials

This study is based on adults of *Megaphragma mymaripenne* Timberlake, 1924 and *Megaphragma amalphitanum* Viggiani, 1997 reared in the laboratory from eggs of *Heliothrips haemorrhoidalis* (Bouché, 1833).

### Scanning electron microscopy (SEM)

The material was fixed in FAE (formaldehyde, acetic acid, ethanol) and stored in 70% ethanol. Skeletal structures were studied using a Jeol JSM-6380 scanning electron microscope following critical point drying (Hitachi HCP-2) and sputter coating of samples with gold (Giko IB-3).

### Histology

For studying internal morphology, material fixed in FAA was dehydrated and embedded in Araldite M. The resulting blocks were cut into complete series of cross sections or longitudinal sections 1 μm thick using a Leica RM2255 microtome. The sections were stained with toluidine blue and pyronine.

### Array tomography

For immunofluorescent staining, the material was fixed in 4% formaldehyde and 0.1 M phosphate buffer and embedded in LR White according to a thermal polymerization protocol [20]. Then the samples were cut into complete series of sections 0.5 μm thick and stained with DAPI. The preparations were studied under an Olympus BX43 microscope with a fluorescent module and a Tucsen TCC-6.1ICE camera.

### Transmission electron microscope (TEM)

The material was fixed in 2% glutaraldehyde solution on 0.1 M cacodylate buffer pH 7.2, post-fixed with 1% osmium tetroxide in the same buffer and en-bloc stained with 1% uranyl-acetate. Specimens were embedded in Epon 812, cut with Leica UC6 ultramicrotomes, stained with lead citrate, and examined with a Jeol JEM-1011 TEM with a Gatan ES500W camera.

### 3D modeling

For 3D computer modeling, series of sections were photographed under a Motic BA410 or Zeiss Axioscope 40 microscope. Following the alignment and calibration of the resulting stack, reconstructions were produced using the program Bitplane Imaris. All structures were segmented manually. The resulting reconstructions were processed using the functions of surface smoothing and rendering in the program Autodesk Maya.

### Nomenclature

The nomenclature follows Hymenoptera Anatomy Ontology [21], Wipfler et al. [22] for the head and Friedrich and Beutel [23] for the thorax, with some additions from Vilhelmsen et al. [24]. The following abbreviations are used in descriptions of muscles: O, origin; I, insertion. The homology of musculature with studies on other hymenopterans is given in the Supplement (S1 and S2 Tables)

## Results

### External morphology

**Fig 1:**
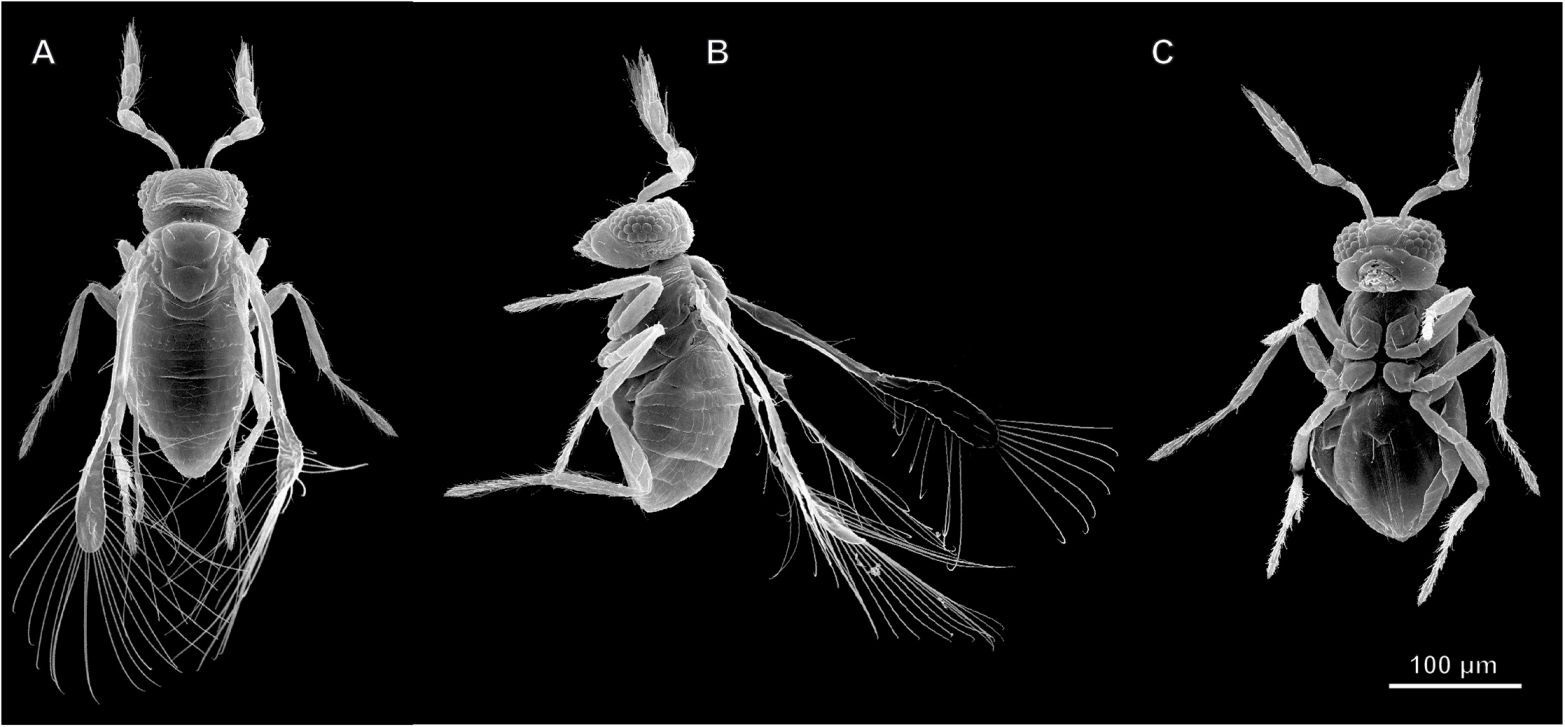
External morphology of *Megaphragma mymaripenne*, SEM. (A) Dorsal view; (B) Lateral view; (C) Ventral view.

Very small body length, 221–255 μm (M = 235, n = 10) in *M*. *mymaripenne* and 232–286 μm (M = 257, n = 10) in *M*. *amalphitanum*. Body compact (Fig 1). Coloration uniform, from yellow to brown, without metallic sheen.

As the two studied species are almost identical in their external and internal morphology, all descriptions refer by default to both of them.

#### Structure of head

**Fig 2:**
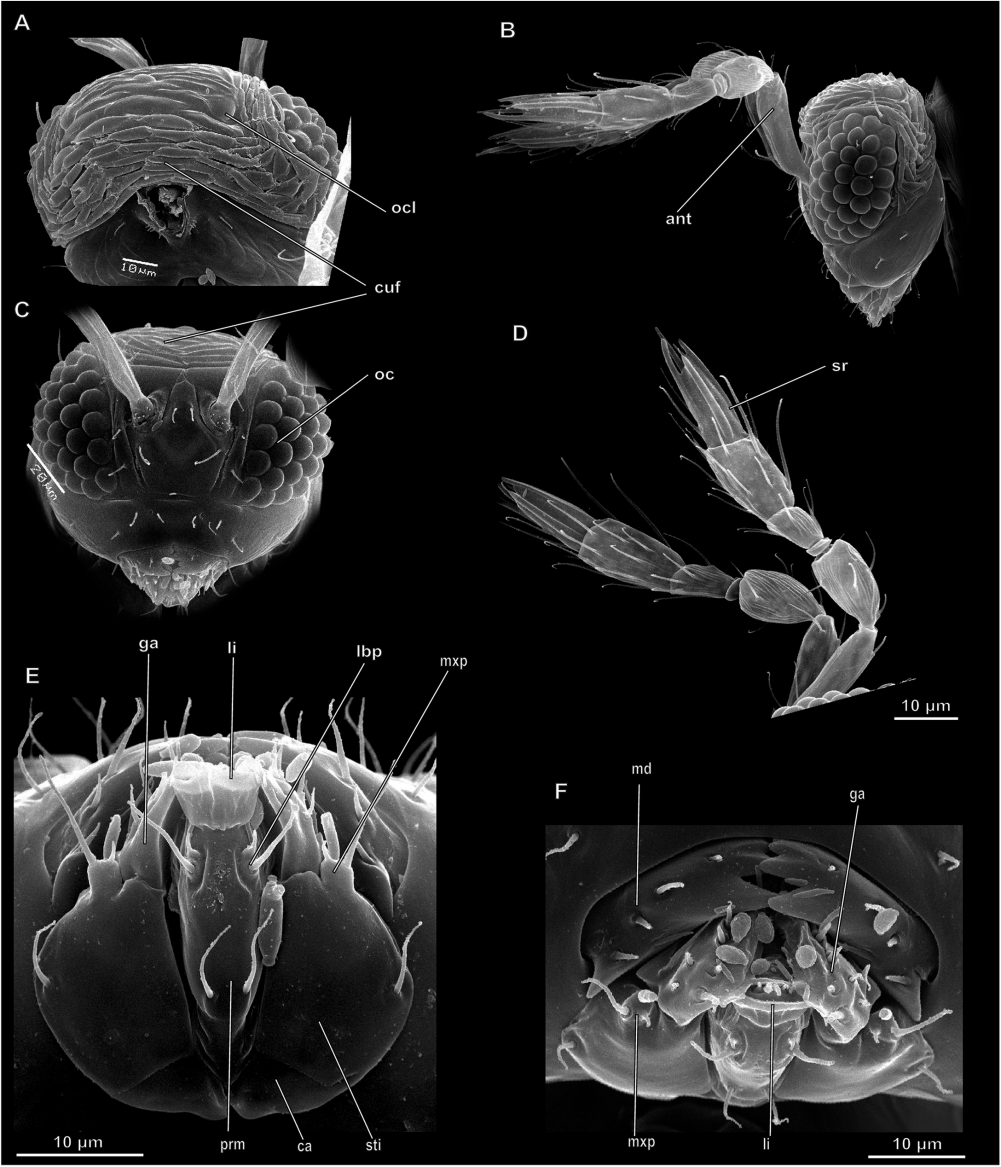
Structure of head in *Megaphragma mymaripenne*, SEM. (A–C) Head; (A) Dorsal view; (B) Lateral view; (C) Frontal view; (D) Antennae; (E) Mouthparts, posterior view; (F) Mouthparts, ventral view; ant–antenna, ca–cardo, cuf–cuticular folds, ga–galea, lbp–labial palp, li–ligula, md–mandible, mxp–maxillary palp, oc–eye, ocl–ocellus, prm–prementum, sr–sensory ridge, sti–stipes.

Head hypognathous, rounded, somewhat flattened in longitudinal direction anteroposteriorly, with posterior surface slightly concave (Fig 2A, 2B and 2C). Head capsule with only one weakly discernible postoccipital ridge and several folds of unclear homology around base of antennae, other sutures or ridges not found. Hypostome and clypeus not distinguishable. Hypostomal bridge absent. Оccipital foramen rather small, keyhole-shaped. Tentorium with anterior and posterior arms, dorsal arms reduced. Tentorial bridge present, laminatentoria absent, Occipital area with numerous folds reducing volume of cranium from pupae to adults.

Compound eyes lateral, consisting of 28–30 ommatidia. Number of ocelli 3.

Antennae 6-segmented, about 150 μm long, consisting of elongate, slightly curved scape, subcylindrical pedicel, small anellus, cylindricl funicle, and 2-segmented club, first antennomere of club widening towards apex, and second antennomere of club narrowing towards apex (Fig 2D).

Mouthparts consisting of labrum, well developed mandibles, maxillae, and labium (Fig 2E and 2F). Labrum weakly developed, represented by rather small triangular membranous plate. Mandibles with undulate medial margin and spines on internal surface. Mola absent. Maxillae combined with labium by membranous septum into labiomaxillary complex. Maxillae consisting of small triangular cardo, broad fusiform stipes, largely fused endite lobes, and palp. Maxillary palp 1-segmented, strongly reduced. Galea bearing large setae and spines, lacinia recognizable as brush of setae. Labium consisting of almost triangular prementum, bearing 1-segmented palps on lateral margin and membranous ligula on apex. Postmentum not identified

#### Structure of mesosoma

Prothorax narrow, consisting of semicircular pronotum and propectus, formed by sternite and pleurites of prothorax. Anterior part of propectus bearing paired cervical processes; head articulating to these processes. Profurca Y-shaped with flattened arms. Pleurite bearing well developed apodeme (propleural arm).

**Fig 3:**
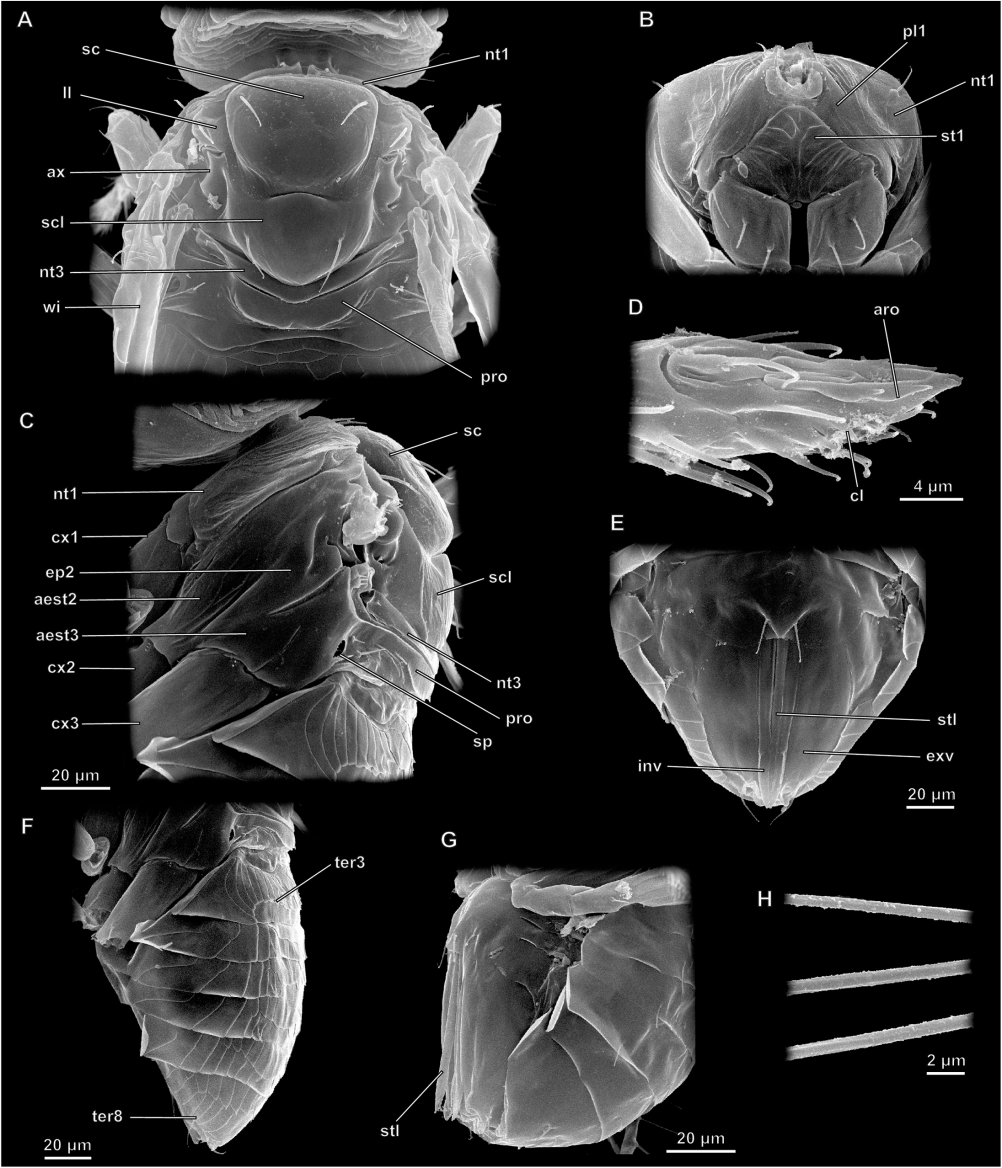
Structure of meso- and of metasoma in *Megaphragma*, SEM. (A–F, H) *M*. *mymaripenne*; (G) *M*. *amalphitanum*; (A–C) Mesosoma; (D) Apex tarsi; (E–G) Metasoma; (H) Wing setae; (A, H) Dorsal view; (B, D) Ventral view; (C, D, F, G) Lateral view; aed–aedeagus, aest2 –mesepistern, aest3 –metepisternum, aro–arolium, ax–axillary sclerite, cl–claw, cx1.2.3 –pro-, meso-, and metacoxae, ep2 –mesepimeron, exv–external valves of ovipositor, inv–internal valves of ovipositor, ll–scapulae, nt1.3 –pro- and metanotum, par–parameres, pl1 –pleurite of prothorax, pre–prepectus, pro–propodeum, prp–propectus, sc–scutum, scl–scutellum, sp–spiracle, stl–stylet of ovipositor, ter–tergite, wi–wing.

Mesothorax distinctly larger than other segments of metasoma (Fig 3A, 3B and 3C). Mesonotum consisting of two parts divided by scuto-scutellar suture. Anterior part divided by longitudinal parapsidal striae into mesoscutum and scapulae (side lobes). Posterior part divided into scutellum and axillae. Lateral part of mesothorax divided into episternum and epimeron by weakly discernible ridge. Prepectus present between pro- and mesothorax. Mesofurca V-shaped, lateral arms well developed. Posterior margin of mesonotum forming mesophargma almost reaching apex of metasoma. Part of mesophragma reaching into metasoma termed postphragma by some authors [9]. Pair of annular uniforous spiracles present between pro- and mesothorax.

Metathorax only represented by narrow semicircular metanotum; other sclerites fused with abdominal segment 1, forming propodeum. Propodeum bearing pair of abdominal spiracles. Metepisterna separated from propodeum by weakly pronounced suture. Epimera fused with propodeum. Pleural apodeme well developed, shaped as high longitudinal ridge with flattened top. Metafurca absent.

**Fig 4:**
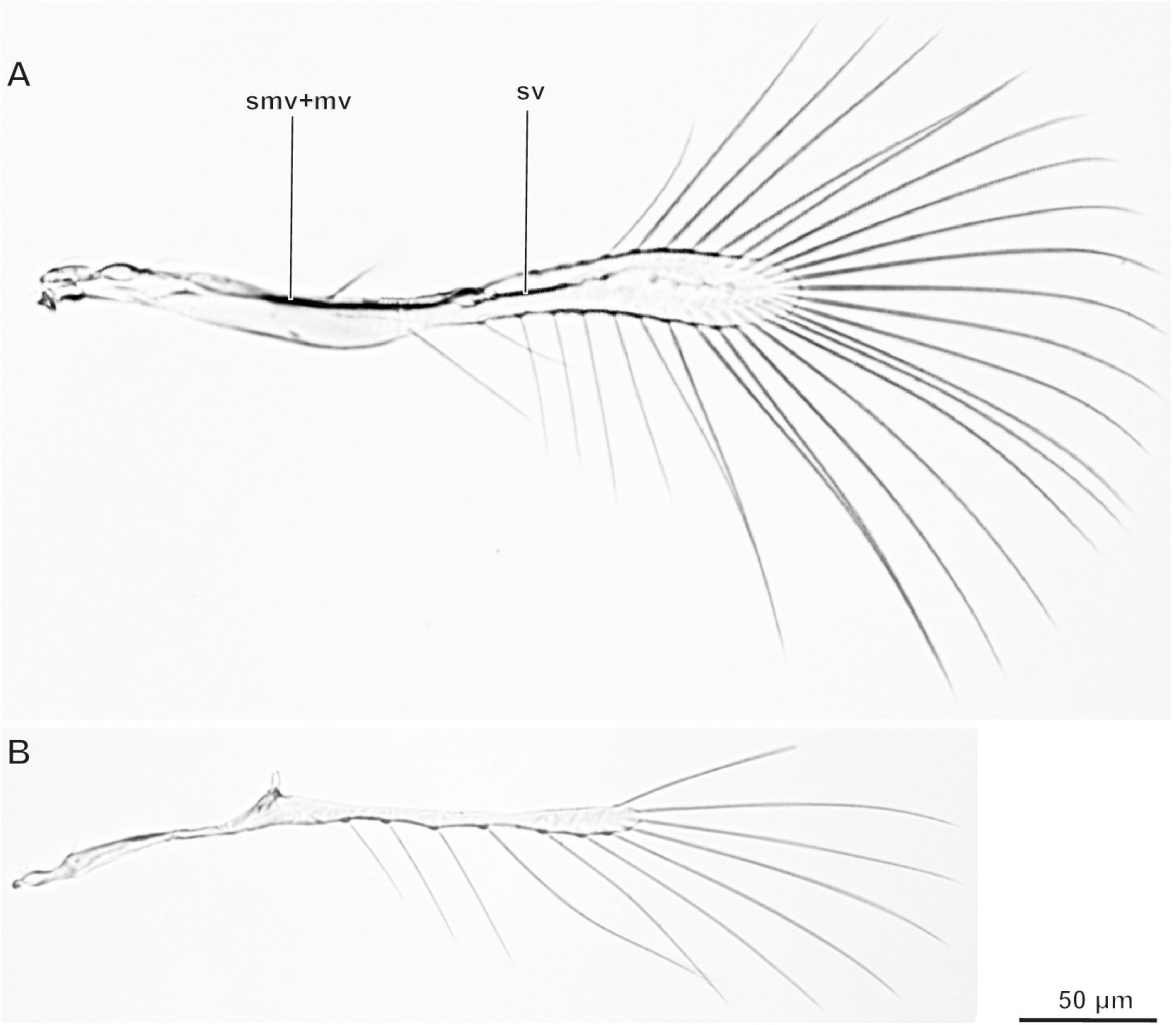
Wings of *Megaphragma mymaripenne*. (A) Forewing; (B) hindwing.

Wings narrow with strongly depleted venation, blade with fringe of long setae on perimeter (Figs 3H and 4). Three veins preserved in forewing: submarginal (Pinto [5]: subcostal and premarginal), marginal, and stigmal (Sorokina [9]: radial), usually fused into one arch near anterior wing margin, formed by fusion of subcosta and radius; the homology of particular parts is discussed in earlier studies [25, 26]. Hind wing narrower than forewing. Hind wing with only one short vein of unknown homology.

Legs slender, ambulatorial, consisting of coxa, 2-segmented trochanter, femur, tibia, and 3-segmented tarsus. Apical tarsomere bearing two claws and well developed arolium (Fig 3D).

#### Structure of metasoma

Petiole indistinct, mesosoma and metasoma broadly joined. Metasoma consisting of six visible tergites, sternites unsclerotized weakly discernible (Fig 3E, 3F and 3G).

### Internal morphology

**Fig 5:**
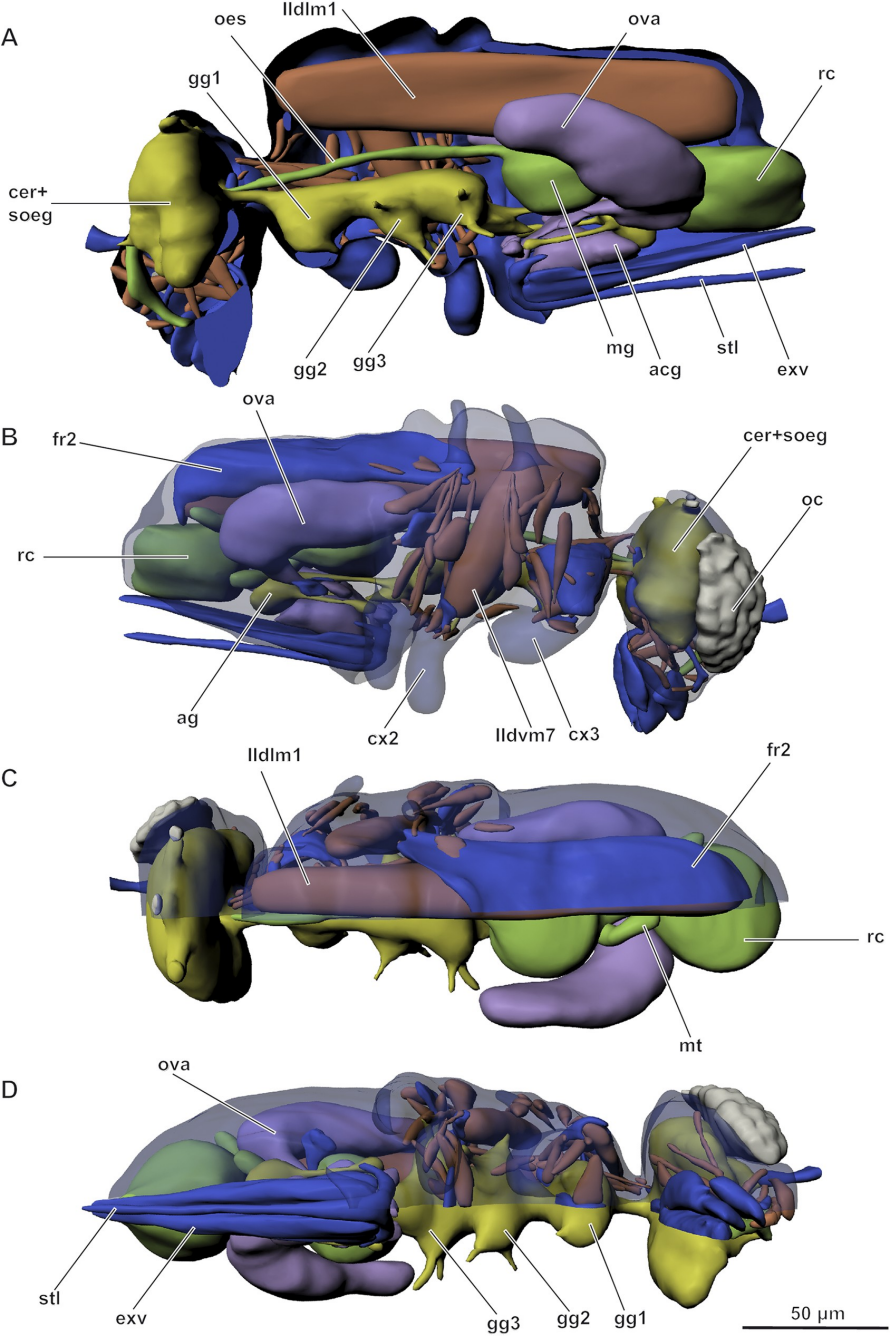
Internal morphology of *Megaphragma mymaripenne*, 3D (for interactive version see S1 Fig). (A) Lateral internal view; (B) Lateral external view; (C) Dorsal view; (D) Ventral view; acg–acid gland, ag–abdominal ganglion, cer–cerebrum, cx1.2 –meso- and metacoxae, exv–external valves of ovipositor, gg1.2.3 –pro-, meso-, and metathoracic ganglia, fr2 –mesophragma; mg–midgut, mt–Malpighian tubules, oc–eye, oes–oesophagus, ova–ovary, rc–rectum, soeg–suboesophageal ganglion, stl–stylet of ovipositor. Colors: blue–cuticle, green–digestive system, yellow–central nervous system, brown–musculature, purple–reproductive system. Musculature see text.

**Fig 6:**
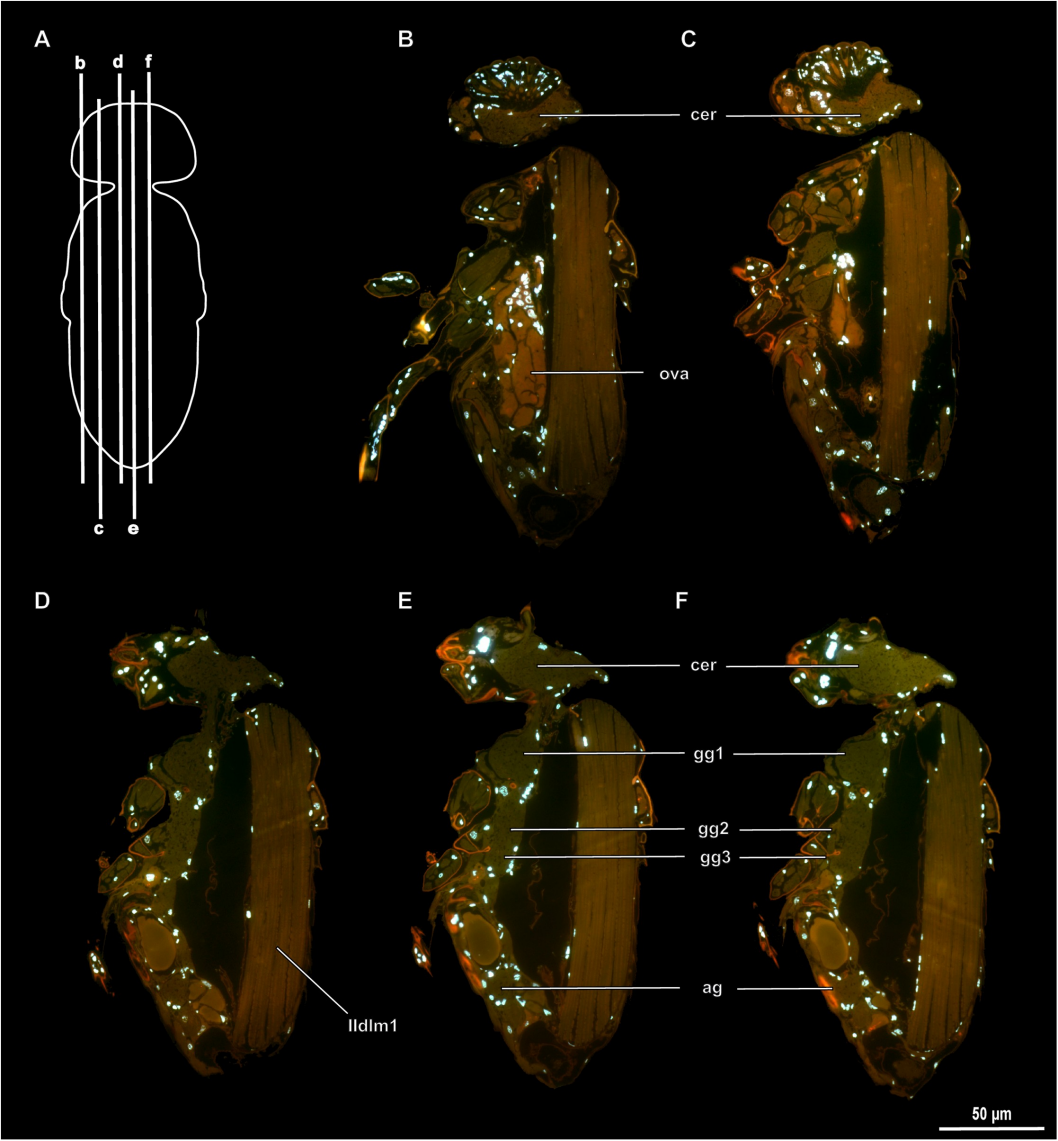
Internal morphology of *Megaphragma amalphitanum*. (A) Scheme of sections, lateral view; (B–F) Longitudinal sections, DAPI and autofluorescence; ag–abdominal ganglion, cer–cerebrum, gg1,2,3 –pro-, meso-, and methatoracic ganglion, ova–ovary. Musculature see text.

General configuration of internal structure: most of head occupied by brain and suboesophageal complex; considerable part of metasoma occupied by musculature; very large muscle (IIdlm1) occupying much of meso- and metasoma; reproductive system occupying most of metasoma (Figs 5 and 6, S1 and S2 Figs).

#### Integument

Integument consisting of cuticle, hypoderm, and basal membrane. Cuticle thickness 0.7–2.4 μm (M = 1.2, n = 80) in *Megaphragma mymaripenne*, thinnest areas of integument being areas between sclerites and pleural regions of mesosoma, and thickest areas being posterior part of head and notal part of mesosoma. Cuticle consisting of epicuticle and procuticle. Procuticle homogeneous. Hypoderm represented by strongly flattened cells up to 1.5 μm thick. Many areas of hypoderm, especially in head, with numerous electron transparent vacuoles.

#### Digestive and excretory systems

Digestive system of generalized type, divided into fore-, mid-, and hindgut (Fig 5, S3 Fig). Fore- and hindgut with thin cuticular lining. Entire gut somewhat longer than body, forming loop in metasoma. Salivary glands absent.

Foregut divided into pharynx, oesophagus, and ingluivies (crop). Oesophagus straight, running through entire mesosoma. Muscles of oesophagus absent. Crop situated in metasoma.

Midgut short, wide. Walls formed by strongly flattened cells. No muscles of midgut found. Peritrophic membrane not found.

Proctodaeum divided into hindgut and rectum.

Boundary between mid- and hindgut bearing three Malpighian tubules, shaped as short slightly curved tubes.

#### Circulatory system and fat body

Circulatory system strongly reduced. Heart and blood vessels absent. Fat body occupying almost all cavities between organs in metasoma and to a smaller degree in mesosoma.

#### Tracheal system

Strongly simplified. Only few tracheae with few branches present, connected to mesosomal and metasomal spiracles. Transverse stems and air sacs absent. Tracheae with structure typical of insects, consisting of hypoderm and intima, intima with helical thickenings (taenidia).

#### Nervous system

Cerebrum and suboesophageal ganglion fused into one and localized entirely in cranium (Fig 5, S3 Fig). Prothoracic ganglion separate, mesothoracic and metathoracic ganglia fused. Abdominal ganglia fused into one synganglion.

**Fig 7:**
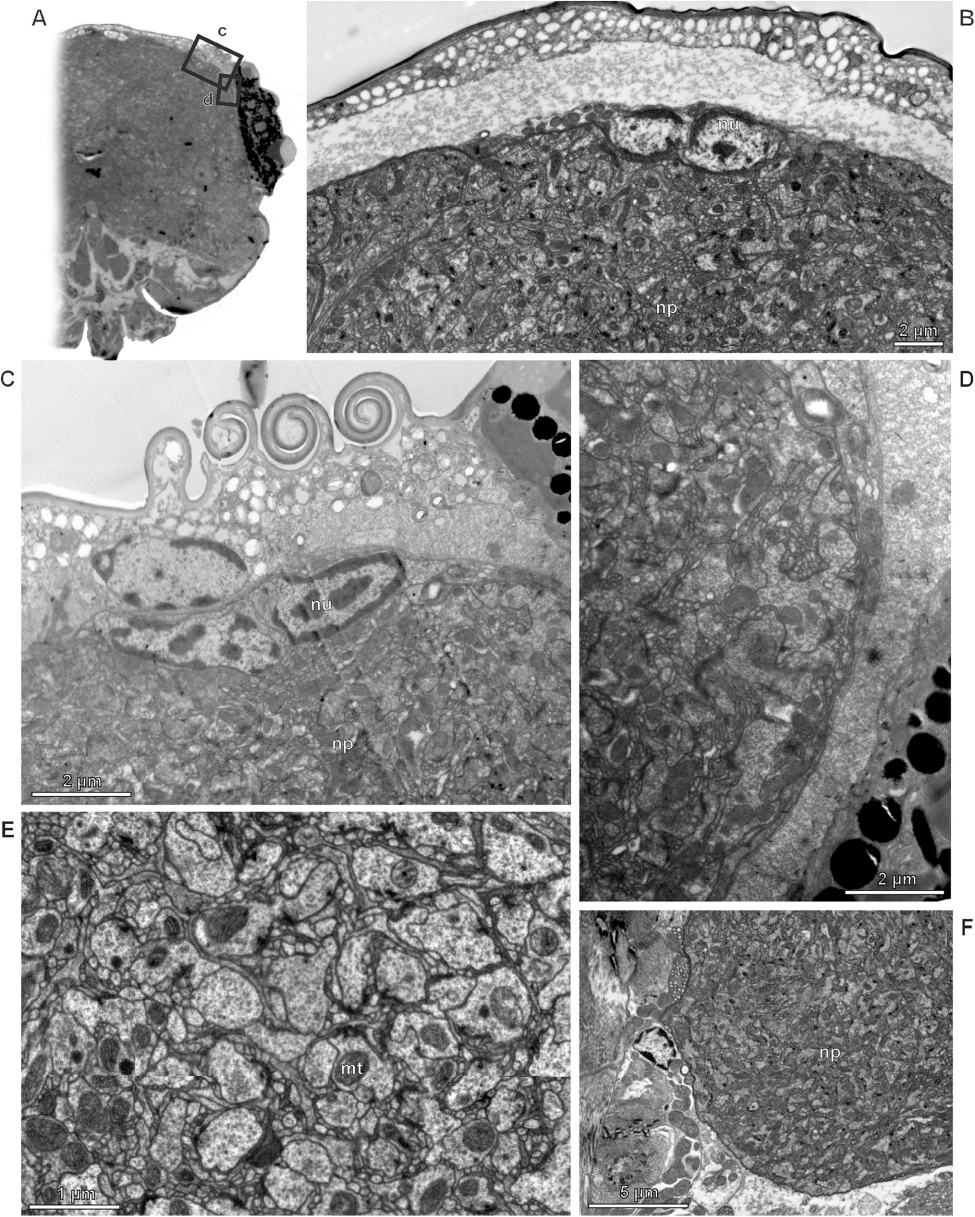
Ultrastructure of the brain in *Megaphragma*, TEM. (A, C, D) *M*. *mymaripenne*; (B, E, F) *M*. *amalphitanum;* mt ‒ mitochondrion, np–neuropil, nu ‒ nucleus.

The central nervous system of *Megaphragma* has a structure fundamentally different from those of all other insects (Figs 6, 7 and S1). It was shown that in adults of *M*. *mymaripenne* the nervous system is almost anucleate, because over 95% of cells in the central nervous system undergo lysis of bodies and nuclei during late stages of the pupal development [2]. In *M*. *amalphitanum* all gangia of the nervous system are also represented almost exclusively by neuropil, which is almost identical in structure to those of larger representatives of related hymenopteran taxa. The optic lobes, central body complex, and antennal lobe are discernible in the neuropil of the brain. At the same time, the central nervous system contains only 320 nuclei, 254 of them in the cerebrum. The pupal nervous systems of both *M*. *amalphitanum* and *M*. *mymaripenne* are almost identical to those of other hymenopterans, and the central nervous system contains about 7000 nuclei, about 4500 of them in the cerebrum.

#### Muscular system

Most muscles of *Megaphragma* are connected with the skeleton via a strongly shortened tonofibril apparatus, which morphologically resembles the desmosome; a similar structure has been described in four-legged mites [27].

**Fig 8:**
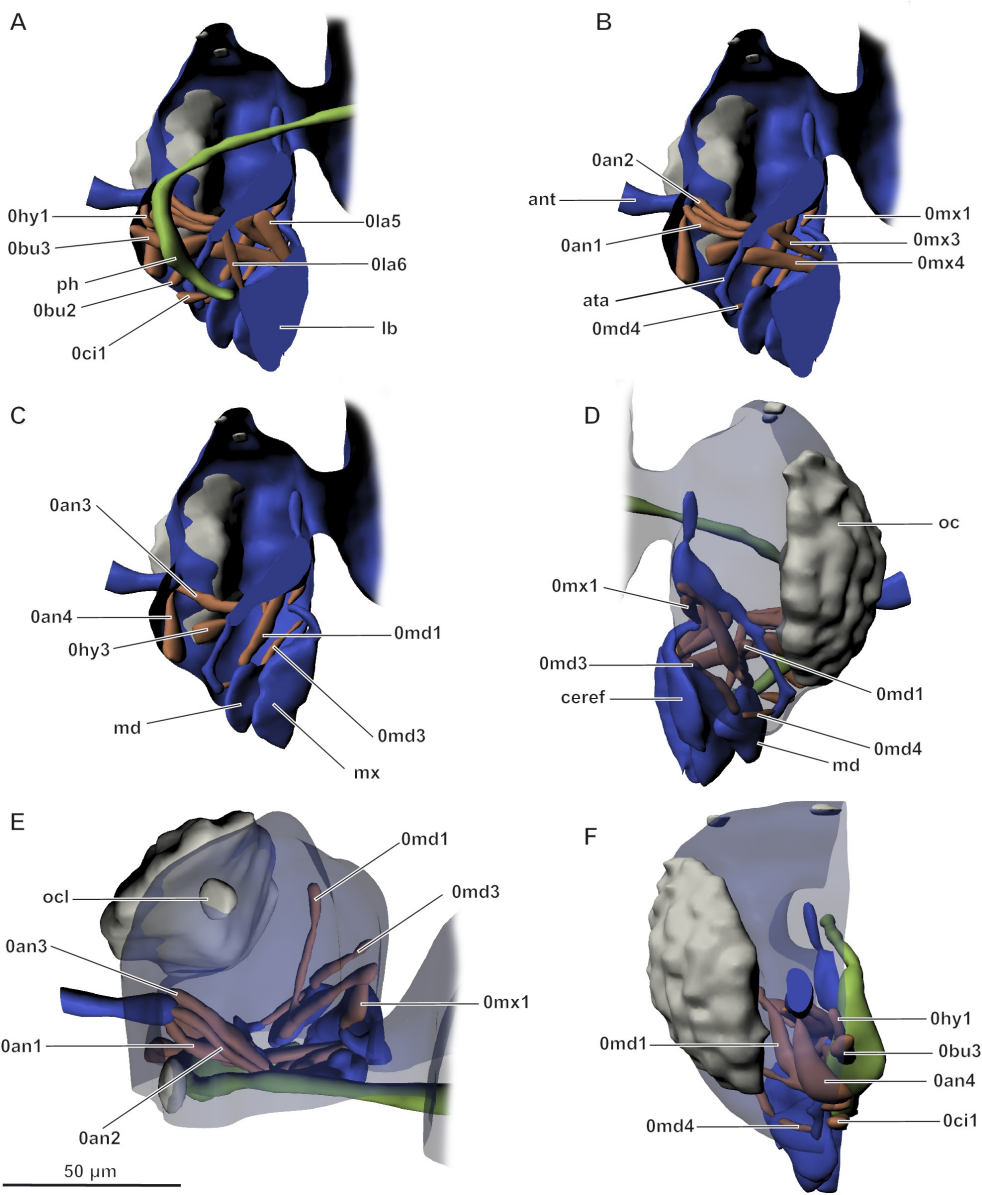
Musculature of head in *Megaphragma mymaripenne*, 3D. (A–C) Lateral internal view; (D) Lateral external view; (E) Dorsal view; (F) Frontal view; ant–antenna, ata–anterior tentorial arms, lb–labium, md–mandible, mx–maxilla, oc–eye, ocl–ocellus, ph–pharynx. Musculature see text.

Musculature of head (Fig 8). 0an1 (M. tentorioscapalis anterior): O, anterior tentorial arms; I, anterior margin of base of scape. 0an2 (M. tentorioscapalis posterior): O, anterior tentorial arms; I, posterior margin of base of scape. 0an3 (M. tentorioscapalis lateralis): O, anterior tentorial arms; I, lateral margin of base of scape. Muscle of unclear homology, possibly 0an4 (M. tentorioscapalis medialis), with atypical attachment site: O, anterolateral part of frons; I, anterior margin of base of scape. 0md1 (M. craniomandibularis internus), consisting of two subunits: O, first, lateral part of cranium; second, gular zone; I, tendon at the median edge of the mandible. 0md3 (M. craniomandibularis externus): O, lateral part of cranium; I, tendon at the lateral edge of the mandible. 0md4 (M. hypopharyngomandibularis): O, anterior tentorial arms; I, lateral edge of mandible. 0mx1 (M. craniocardinalis): O, posterior part of cranium; I, ventrolateral part of cardo. 0mx3 (M. tentoriocardinalis): O, anterior tentorial arms; I, cardo. 0mx4 (M. tentoriostipitalis anterior): O, anterior tentorial arms; I, base of stipes. 0la5 (M. tentoriopraementalis): O, anterior tentorial arms; I, posterior margin of prementum. 0la6 (M. tentorioparaglossalis): O, anterior tentorial arms; I, anterior margin of prementum. 0hy1 (M. frontooralis): O, frons; I, posterior margin of epipharynx. 0hy3 (M. craniohypopharyngealis): O, anterior tentorial arms; I, anterior margin of epipharynx. 0ci1 (M. clypeopalatalis): O, clypeus; I, dorsal part of epipharynx. 0bu2 (M. frontobuccalis anterior): O, frons; I, dorsolateral part of pharynx. 0bu3 (M. frontobuccalis posterior): O, frons; I, dorsal part of pharynx. 0st1 (M. annularis stomodaei): transverse musculature, developed only on dorsal surface of pharynx. Internal musculature of antennae and mouthparts not studied because of extremely small size.

**Fig 9:**
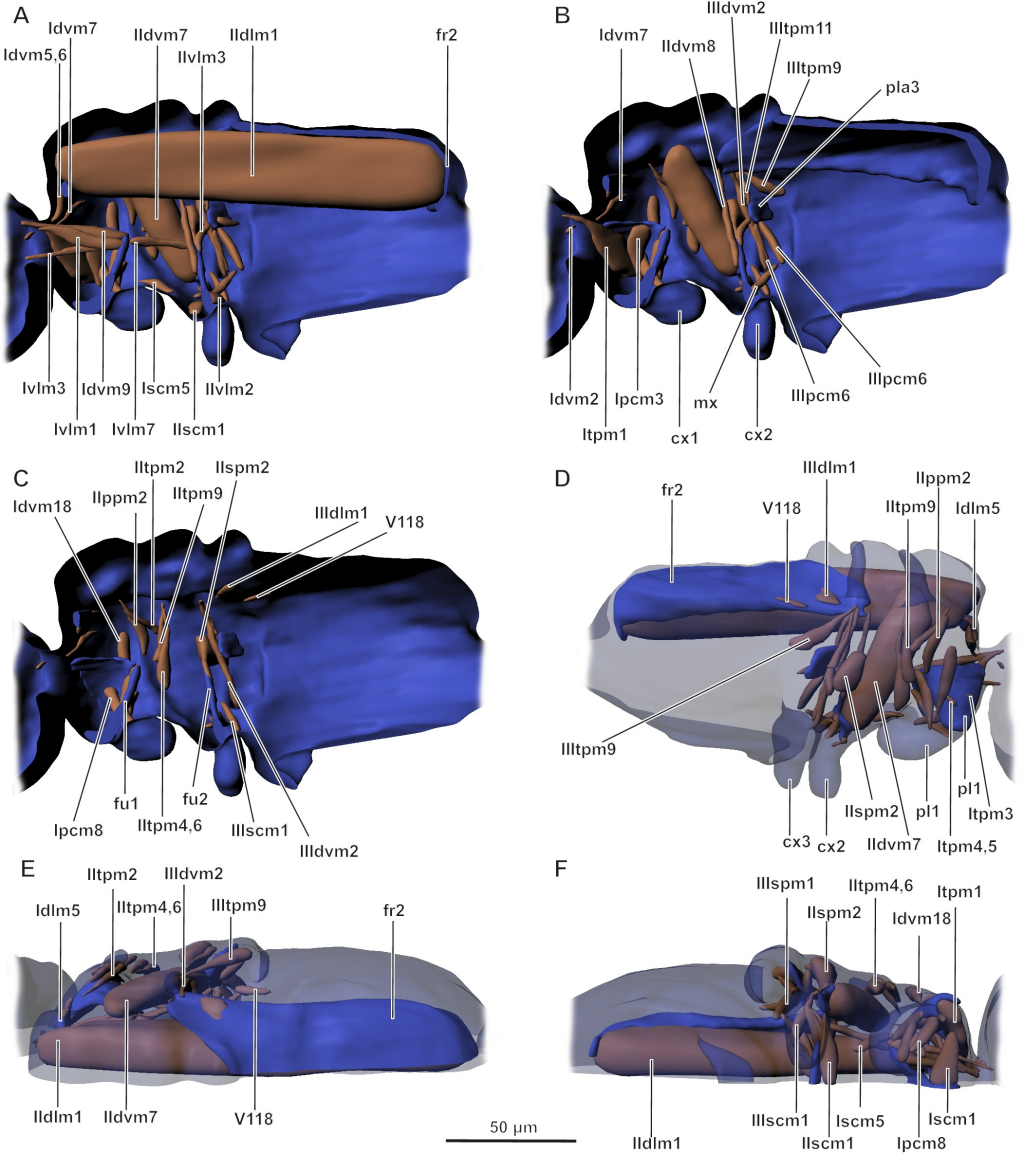
**Musculature of mesosoma in *Megaphragma mymaripenne*, 3D/** (A–C) Lateral internal view; (D) Lateral external view; (E) Dorsal view; (F) Ventral view; pl1 –pleurite of prothorax, pla3 –pleural apodeme of metathorax, fr1.2 –pro- and mesophragma, fu1.2 –pro- and mesofurca, cx1.2.3 –pro-, meso-, and metacoxae. Musculature see text.

Musculature of mesosoma (Fig 9). Prothorax. Idlm5 (M. pronoto-phragmalis anterior): O, prophragma; I, medial part of pronotum. Idvm2 (M. cervico-occipitalis medialis): O, cervical region; I, occipital region. Itpm3 (M. pronoto-pleuralis anterior): O, lateral part of notum; I, propleurite, could not be determined precisely. Idvm5 (M. pronoto-cervicalis anterior) and Idvm6 (M. pronoto-cervicalis medialis) fused: O, posterior part of pronotum and prophragma; I, cervical region. Idvm7 (M. pronoto-cervicalis posterior): O, pronotum; I, cervical region. Idvm9 (M. profurca-occipitalis): O, profurca; I, occipital region. Idvm18 (M. pronoto-coxalis lateralis): O, pronotum; I, lateral margin of base of coxa. Itpm2 (M. propleuro-occipitalis): O, occipital zone; I, pleural apodeme. Itpm4-5 (M. pronoto-apodemalis): O, lateral part of pronotum; I, pleurite. Ipcm3 (M. propleuro-trochantinalis): O, pleural apodeme; I, trochantin. Ipcm4 (M. propleuro-coxalis superior): O, pleural apodeme; I, anterior margin of base of coxa. Ipcm8 (M. propleuro-trochanteralis): O, pleural apodeme; I, trochanter, via fine tendon. Ivlm1 (M. profurca-cervicalis): O, profurca; I, cervical region. Ivlm3 (M. profurca-tentorialis): O, profurca; I, postoccipital region. Ivlm7 (M. profurca-mesofurcalis): O, profurca; I, mesofurca. Iscm1 (M. profurca-coxalis anterior): O, profurca; I, anterior margin of base of coxa. Iscm2 (M. profurca-coxalis posterior): O, profurca; I, posterior margin of base of coxa. Iscm3 (M. profurca-coxalis medialis): O, profurca; I, medial margin of base of coxa. Iscm5 (M. prospina-coxalis): O, fold between pro- and metathorax; I, posterior margin of base of coxa. Internal muscles of legs not studied because of extremely small size.

Mesothorax. IIdlm1 (M. prophragma-mesophragmalis) largest muscle: O, prophragma; I, mesophragma. IIdvm7 (M. mesonoto-trochanteralis): O, mesonotum; I, apodeme of trochanter. IIdvm8 (M. mesofurca-phragmalis): O, mesofurca; I, mesophragma. IItpm2 (M. mesopleura-praealaris): O, pleurite; I, prealar zone. IItpm4 (M. mesonoto-pleuralis anterior): fused with IItpm6, O, pleurite; I, margin of mesonotum. IItpm6 (M. mesonoto-pleuralis posterior) fused with IItpm4. IItpm9 (M. mesepimero-axillaris tertius): O, pleurite; I, third axillary plate. IIppm2 (M. mesobasalare-intersegmentalis): O, intersegmental juncture; I, basalare. IIspm2 (M. mesofurca-pleuralis): O, apex of mesofurca, I, pleurite. Muscle of unclear homology, possibly, IIvlm3 with atypical attachment site: O, mesofurca; I, fold between meso- and metasoma. IIscm1 (M. mesofurca-coxalis anterior): O, mesofurca; I, anterior margin of base of coxa. IIscm2 (M. mesofurca-coxalis posterior): O, mesofurca; I, posterolateral margin of base of coxa. IIscm3 (M. mesofurca-coxalis medialis): O, mesofurca; I, medial margin of base of coxa. Internal muscles of legs not studied because of extremely small size.

Metathorax and propodeum. IIIdlm1 (M. mesophragma-metaphragmalis): O, mesophragma; I, metaphragma. IIIdvm2 (M. metanoto-trochantinalis anterior): O, metanotum; I, trochanter. IIItpm5 (M. metanoto-pleuralis medialis) and IIItpm6 (M. metanoto-pleuralis posterior) fused: O, pleural apodeme of metathorax; I, lateral margin of metanotum. IIItpm9 (M. metepimero-axillaris tertius): O, pleural apodeme; I, third axillary plate. IIItpm7 (M. metanepisterno-axillaris): O, pleural apodeme; I, third axillary plate. IIItpm11 (M. metapleura-subalaris): O, pleural apodeme; I, subalare. IIIspm1 (M. metapleura-sternalis): O, ventral part of propodeum; I, basalare. IIIpcm3 (M. metanepisterno-coxalis anterior): O, pleural apodeme; I, anterolateral margin of base of coxa. IIIpcm4 (M. metanepisterno-coxalis posterior): O, pleural apodeme; I, posterolateral margin of base of coxa. IIIpcm6 (M. metapleura-trochanteralis): O, pleural apodeme; I, trochanter. IIIscm1 (M. metafurca-coxalis anterior): O, fold between meso- and metathorax; I, anterior margin of base of coxa. Muscle V118, described only in hymenopterans (Vilhemsen et al. [24]: no. 118, ph3-T2. M. metaphragma-second abdominal tergal): O, metaphragma; I, tergite of abdominal segment 2. Additional muscle of unclear homology (mx): O, base of mesofurca; I, medial margin of base of metacoxae. Internal muscles of legs not studied because of extremely small size.

Musculature of metasoma. Dorsal longitudinal muscles (Mm. dorsales): O, anterior phragma; I, posterior phragma. Ventral longitudinal muscles (Mm. ventrales), several parallel fibers: O, anterior margin of segment; I, posterior margin of segment. Dorsoventral muscles, several of urotergosternal muscles, homology unknown. Ovipositor with group of strong retractors (Snodgrass [28]: no. 198, 199).

#### Reproductive system

Male reproductive system consisting of paired testes, paired vasa deferentia, ductus ejaculatorius, accessory glands, and copulatory apparatus (S4 Fig). **External male genitalia** represented by simple aedeagus, phallobase, and parameres.

Female reproductive system consisting of paired ovaries and paired oviducts fused into unpaired oviduct connected to vagina (S4 Fig). Well developed acid gland, alkaline gland, and paired accessory glands present. Each ovary consisting of 2 polytrophic ovarioles. **Ovipositor** consisting of outer ovipositor plates (derivates of tergite 9), inner ovipositor plates (Val3), sheath (fused Val2), and stylets (Val1). Spermatheca rather small, rounded.

## Discussion

Although the principal purpose of this study is to analyze the effects of miniaturization on the body structure in the smallest insects, investigations into the anatomy of *Megaphragma* and *Trichogramma* revealed several features that are potentially useful for the macrotaxonomy of Chalcidoidea. These features are three apomorphic characters of the musculature of Trichogrammatidae: the absence of muscles 0lb2 and IIdvm1 and the hypertrophy of IIdlm1. Other derived features are the related elongation of the mesophragma to the middle part of the metasoma (*Trichogramma*) or even the apical region (*Megaphragma*), and the unique absence of the heart in *Trichogramma* and *Megaphragma*.

Problems related to insect miniaturization have been studied and discussed rather intensely over the last few years [1], but the new data on the morphology of *Megaphragma* considerably supplement our notions of the phenomenon of miniaturization in insects. The genus *Megaphragma* includes some of the smallest insects, *M*. *mymaripenne* and *M*. *amalphitanum*, the smallest insects the anatomy of which has been described in detail to date. This makes them unique subjects for discussions of miniaturization in insects.

In general, the external morphology and skeletal structure of *Megaphragma* shows no considerable deviations from the morphology of other Chalcidoidea, except for the few peculiar features described below. *Megaphragma* has no suture on the head capsule and only one ridge. Many other microinsects also display reductions in the number of sutures, which are sometimes completely absent [1]. The tentorium of *Megaphragma* differs from those of the majority of large hymenopterans in the absence of the dorsal arms. They are also absent in *Trichogramma* [10], the mymarid *Anaphes* [29], and many miniaturized beetles [30, 31, 32, 33]. A peculiar feature of Trichogrammatidae is the hypertrophy of the mesophragma, which is deeply sunk into the body and reaches the middle of the metasoma in *Trichogramma* and almost the apex of the metasoma in *Megaphragma*. In addition, *Megaphragma* lacks the metafurca, a reduction that has not been described for adults of any other microinsect. However, the first instar larva of the strepsipteran *Mengenilla chobauti* lacks the entire endoskeleton of the mesosoma [34]. In spite of the absence of the metafurca, the hindlegs function normally (personal observation of this author). The wings of *Megaphragma* display pronounced ptiloptery, which is typical of most microinsects [1]. The sternites of the metasoma in *Megaphragma* are weakly sclerotized and almost indiscernible, which distinguishes it from *Trichogramma* and other Chalcidoidea [10], and also from adults of most other microinsects [1].

The internal morphology of *Megaphragma*, on the one hand, retains complexity in spite of the extremely small size of the body, and on the other hand, demonstrates some peculiar features related to miniaturization.

The cuticle in *Megaphragma* is considerably thinner than in large representatives of related groups of insects but similar to the thickness of the cuticle in other microinsects [30, 35, 36]. The procuticle of both *Megaphragma* and *Trichogramma* is not differentiated into the exo- and endocuticle [10].

*Megaphragma* lacks all muscles of the midgut, which are absent also in many other microinsects [1]. *Megaphragma* and *Trichogrmma* lack salivary glands, which distinguishes them from the other chalcidoids [10]. *Megaphragma* has only three Malpighian tubules, as in *Anaphes* [29] or *Trichogramma* [10], and they are fewer than those of large hymenopterans (which have up to 50 Malpighian tubules).

The tracheal system of *Megaphragma* is strongly reduced (as in other microinsects), compared to large representatives of related groups [1].

The heart and blood vessels have not been found either in *Megaphragma* or in *Trichogramma*, in contrast to *Anaphes* and other chalcidoids [29]. Absence of the heart has been described in Ptiliidae, and it was proposed earlier that diffusion is sufficient for the transportation of substances at such a small size of the body [30, 35, 37].

*Megaphragma* displays oligomerization and concentration of ganglia (the suboesophageal ganglion and prothoracic ganglion are not fused, the mesothoracic ganglion and metathoracic ganglion are fused, and the abdominal ganglia are fused but not shifted into the mesosoma). Other microhymenopterans, thrips, and psocopterans display less pronounced oligomerization and concentration of ganglia of the central nervous system [10, 38]. In microcoleopterans all ganglia (including those of the head and metasoma) are more strongly fused or concentrated and partly or completely shifted into the mesosoma [31, 35]. The number of neurons in *Megaphragma* is strongly reduced, as in all other studied microinsects [15, 39]. Adults of the two studied species of *Megaphragma* display the unique phenomenon of reduced number of nuclei and cell bodies of neurons, as a result of lysis at later stages of pupal development. *Megaphragma* displays a considerable decrease in the number of ommatidia without considerable changes in the size of the ultrastructural organization of particular ommatidia, as in other insects [16, 17].

The set of muscles in *Megaphragma* is somewhat smaller than in other minute hymenopterans and smaller by up to 20% than in some large representatives of related taxa (S3 Table). The head contains 18 muscles in *Megaphragma* and 19 muscles in *T*. *evanescens*, but 20 in *Anaphes* and *Hemiptarsenus* [10]. The mesosoma contains 45 pairs of muscles in *Megaphragma*, 50 in *Trichogramma*, 50–51 in Mymaridae, 51–56 in other representatives of Chaicidoidea, and 53–55 in Ichneumonoidea [10, 24]. However, analysis of peculiar features of the musculature found in different groups reveals not a single reduction shared by all groups of microinsects and only three changes shared by several groups: the absence of 0hy9 and 0st2 in adults of Ptiliidae and Corylophidae and absence of IItpm10 in adults of Corylophidae and *Megaphragma*. A unique feature of Trichogrammatidae is the absence of the muscle IIdvm1 (M. mesonoto-sternalis), one of the principal flight muscles and one of the largest muscles found in all studied hymenopterans [24]. The problem of the hypertrophy of the muscle IIdlm1 requires further study.

Gonads in *Megaphragma* are paired, as in *Trichogramma* and *Anaphes*. This feature distinguishes hymenopterans from Ptiliidae, in which the gonads are reduced on one side [30, 35, 37].

## Conclusions

Strong simplification of structure, hypothesized and termed pumilic degeneration in a theoretical study by Gorodkov [40], is not found in *Megaphragma*, or, indeed, in most microinsects [41, 42]. Many of the peculiar miniaturization-related features of structure found in *Megaphragma*, are also found in other microinsects (ptiloptery, reduction of the number of fully formed elements of the skeleton, oligomerization and condensation of the central nervous system, absence of the heart, etc.), but some such features (lysis of cell bodies and nuclei of neurons at late stages of pupal development, absence of metafurca and several muscles) are unique and have not been found in any other studied insect.

## Supporting information

S1 Fig Interactive animated 3D reconstruction of the Megaphragma mymaripenne for Fig 5. Click on the figure to start interactive 3D view. Colors: blue–cuticle, green–digestive system, yellow–central nervous system, brown–musculature, purple–reproductive system.
(PDF)

S2 Fig Internal structure of *Megaphragma mymaripenne*. (A) Scheme of sections, lateral view; (B–E) Longitudinal sections, toluidine blue, pyronine; acg–acid gland, ag–abdominal ganglion, cer–cerebrum, fr2 –mesophragma, gg1,2,3 –pro-, meso-, and metathoracic ganglion, mg–midgut, mt–Malpighian tubules, oc–eye, ova–ovary, rc–rectum. Musculature see text.
(PDF)

S3 Fig Internal morphology of *Megaphragma mymaripenne*, 3D. (A, B) Intestine and Malpighian tubules; (C, D) Central nervous system; (A, C) Dorsal view; (B, D) Lateral view; ag–abdominal ganglion, cer–cerebrum, gg1.2.3 –pro-, meso-, and metathoracic ganglia, mg–midgut, mt–Malpighian tubules, oes–oesophagus, rc–rectum, soeg–suboesophageal ganglion.
(PDF)

S4 Fig Reproductive system of *Megaphragma mymaripenne*, 3D. (A, B) Female; (C, D) Male; (A, C) Lateral view; (B, D) Dorsal view; acg–acid gland, aed–aedeagus, agl–accessory glands, alg–alkaline gland, ova–ovary, spt–spermatheca, stl–stylet of ovipositor, te–testis, val–valves of ovipositor.
(PDF)

S1 Table Homology of head musculature in Hymenoptera. (PDF)

S2 Table Homology of musculature of mesosoma in Hymenoptera. (PDF)

S3 Table Musculature of adult Chalcidoidea and Ichneumonoidea. (PDF)

S1 File Supplementary references. (PDF)
