# Supplementary material for: Anatomy of adult Megaphragma (Hymenoptera: Trichogrammatidae), one of the smallest insects, and new insight into insect miniaturization
Source: PLoS One. 2017 May 3;12(5):e0175566. doi: 10.1371/journal.pone.0175566 (PMC5414980; doi:10.1371/journal.pone.0175566)
Supplement: S1 Fig — Click on the figure to start interactive 3D view. Colors: blue–cuticle, green–digestive system, yellow–central nervous system, brown–musculature, purple–reproductive system. (PDF) [file pone.0175566.s001.pdf]

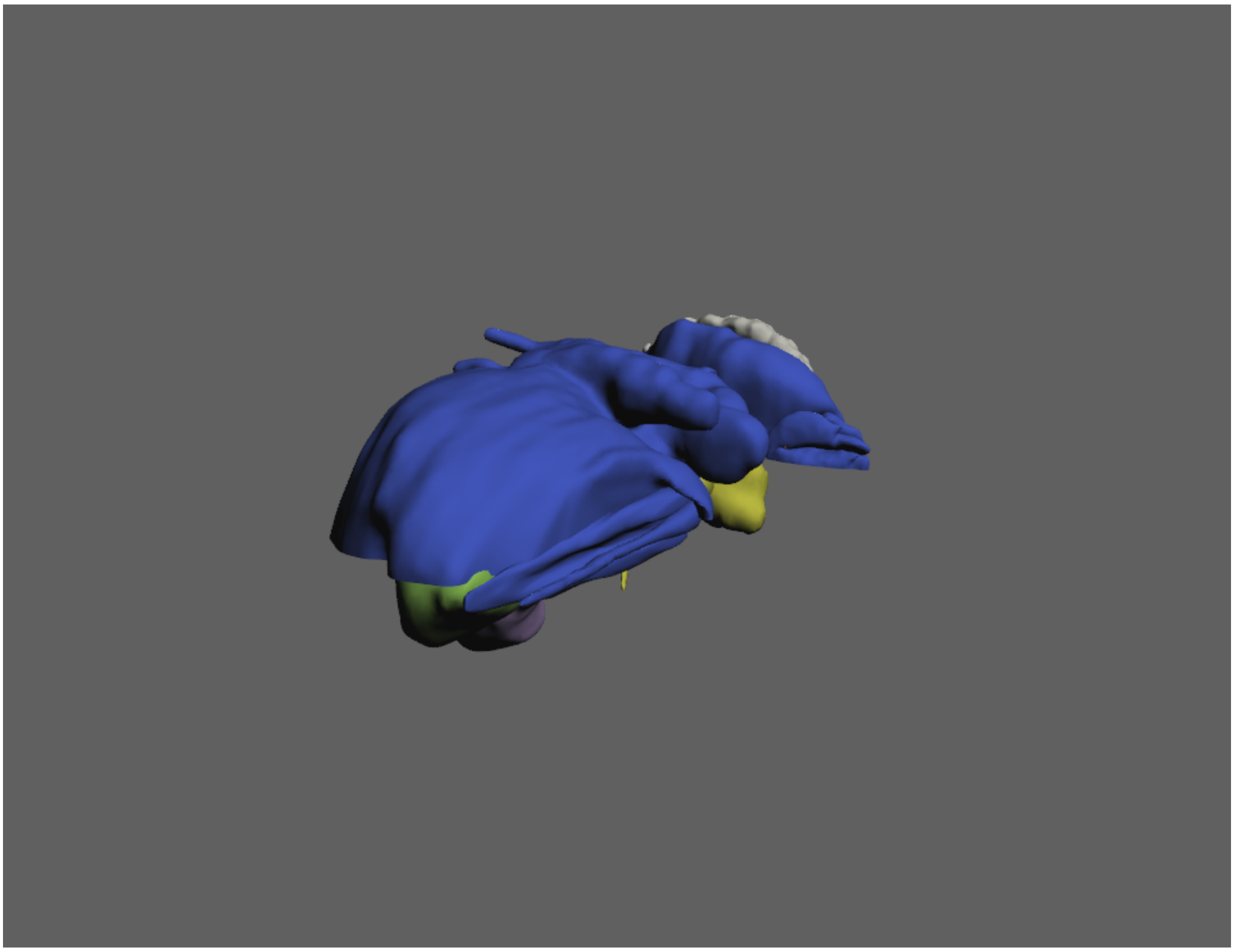

S1 Fig. Interactive animated 3D reconstruction of the *Megaphragma mymaripenne* for Fig 5. Click on the figure to start interactive 3D view. Colors: blue – cuticle, green – digestive system, yellow – central nervous system, brown – musculature, purple – reproductive system.
