# Supplementary material for: Anatomy of adult Megaphragma (Hymenoptera: Trichogrammatidae), one of the smallest insects, and new insight into insect miniaturization
Source: PLoS One. 2017 May 3;12(5):e0175566. doi: 10.1371/journal.pone.0175566 (PMC5414980; doi:10.1371/journal.pone.0175566)
Supplement: S2 Fig — (A) Scheme of sections, lateral view; (B–E) Longitudinal sections, toluidine blue, pyronine; acg–acid gland, ag–abdominal ganglion, cer–cerebrum, fr2 –mesophragma, gg1,2,3 –pro-, meso-, and metathoracic ganglion, mg–midgut, mt–Malpighian tubules, oc–eye, ova–ovary, rc–rectum. Musculature see text. (PDF) [file pone.0175566.s002.pdf]

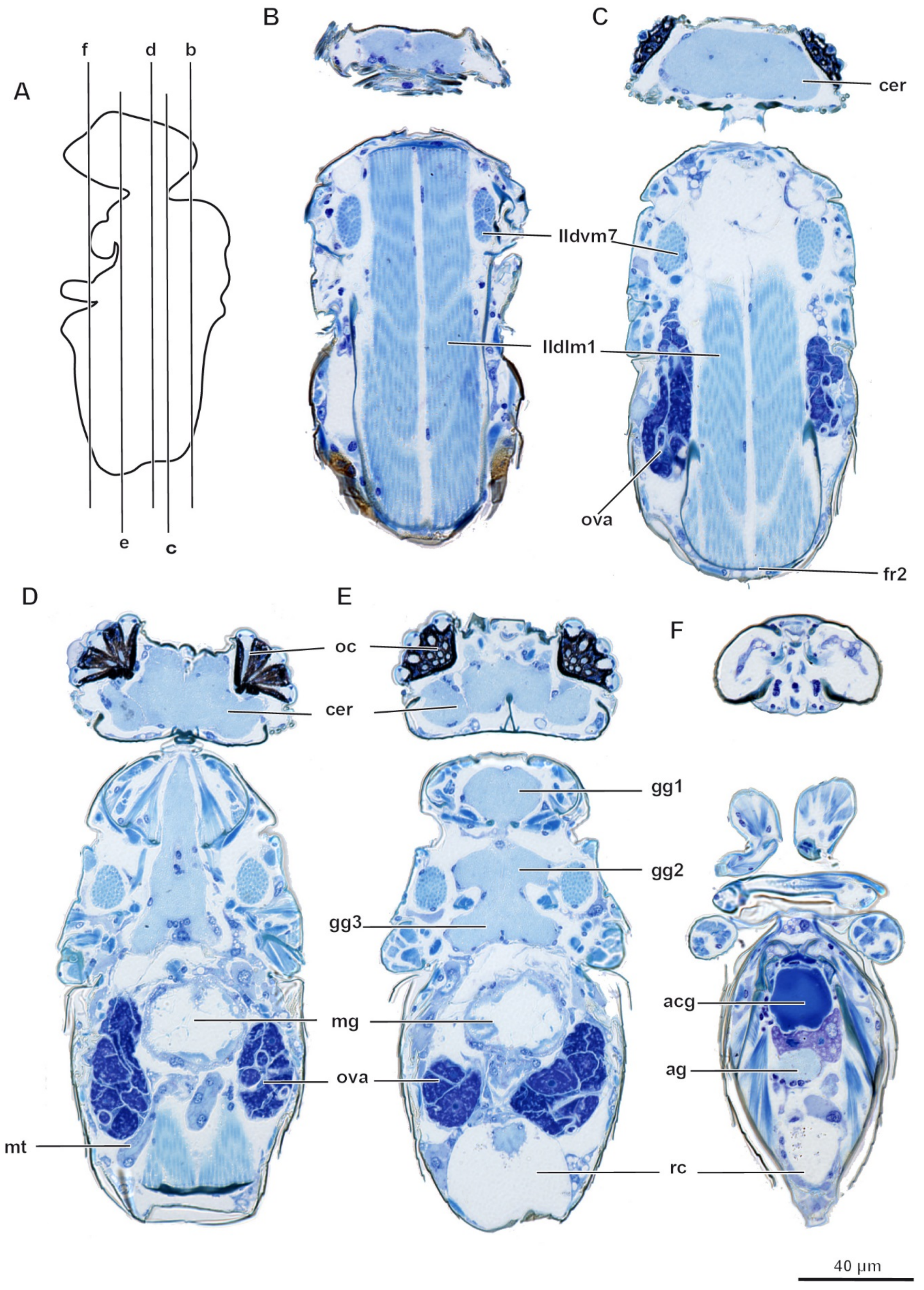

S2 Fig. Internal structure of *Megaphragma mymaripenne*.

(A) Scheme of sections, lateral view; (B–E) Longitudinal sections, toluidine blue, pyronine; acg – acid gland, ag – abdominal ganglion, cer – cerebrum, fr2 – mesophragma, gg1,2,3 – pro-, meso-, and metathoracic ganglion, mg – midgut, mt – Malpighian tubules, oc – eye, ova – ovary, rc – rectum. Musculature see text.
