# Supplementary material for: Anatomy of adult Megaphragma (Hymenoptera: Trichogrammatidae), one of the smallest insects, and new insight into insect miniaturization
Source: PLoS One. 2017 May 3;12(5):e0175566. doi: 10.1371/journal.pone.0175566 (PMC5414980; doi:10.1371/journal.pone.0175566)
Supplement: S3 Fig — (A, B) Intestine and Malpighian tubules; (C, D) Central nervous system; (A, C) Dorsal view; (B, D) Lateral view; ag–abdominal ganglion, cer–cerebrum, gg1.2.3 –pro-, meso-, and metathoracic ganglia, mg–midgut, mt–Malpighian tubules, oes–oesophagus, rc–rectum, soeg–suboesophageal ganglion. (PDF) [file pone.0175566.s003.pdf]

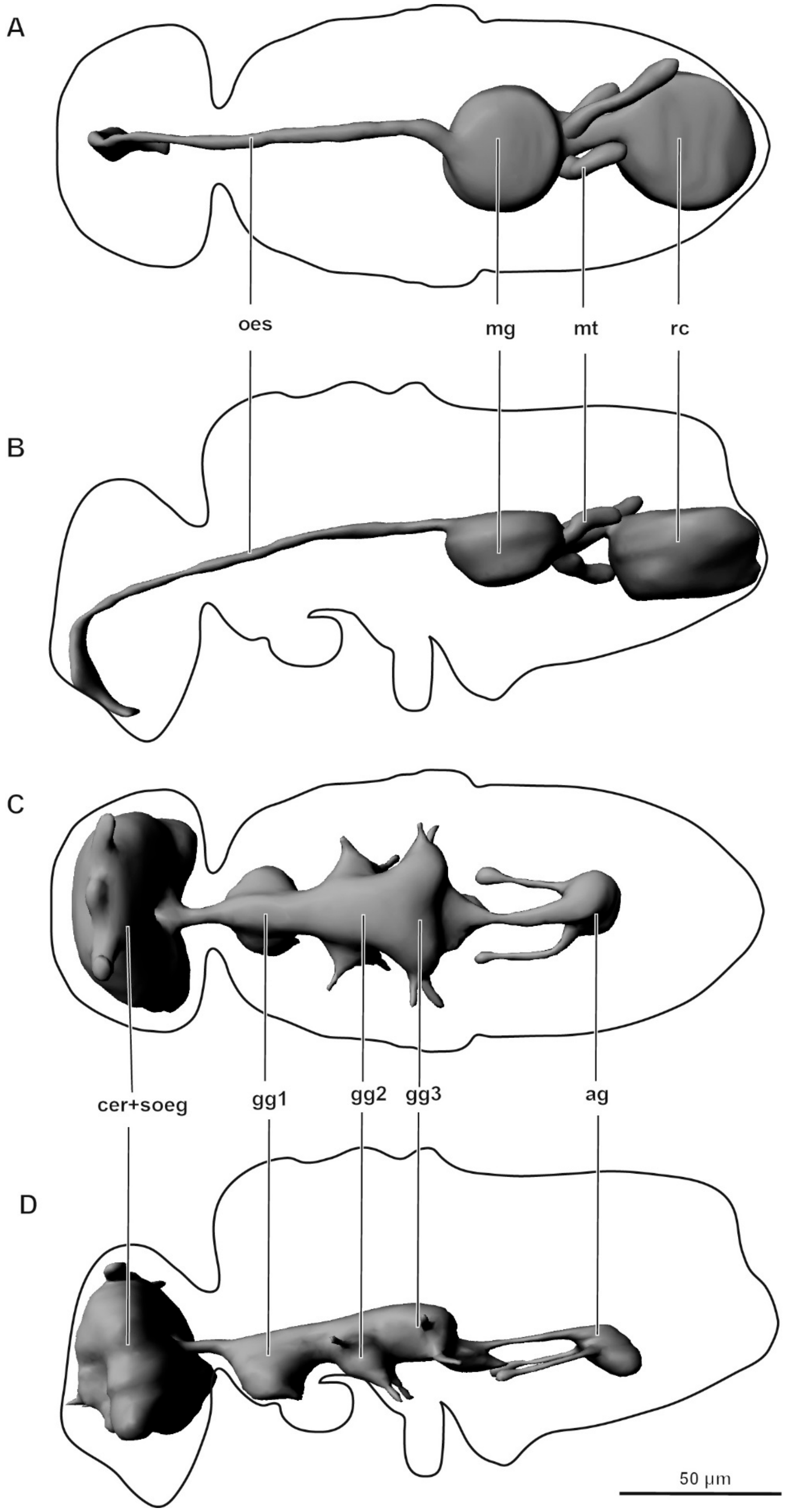

S3 Fig. Internal morphology of *Megaphragma mymaripenne*, 3D.

(A, B) Intestine and Malpighian tubules; (C, D) Central nervous system; (A, C) Dorsal view; (B, D) Lateral view; ag – abdominal ganglion, cer – cerebrum, gg1.2.3 – pro-, meso-, and metathoracic ganglia, mg – midgut, mt – Malpighian tubules, oes – oesophagus, rc – rectum, soeg – suboesophageal ganglion.
