# Supplementary material for: Anatomy of adult Megaphragma (Hymenoptera: Trichogrammatidae), one of the smallest insects, and new insight into insect miniaturization
Source: PLoS One. 2017 May 3;12(5):e0175566. doi: 10.1371/journal.pone.0175566 (PMC5414980; doi:10.1371/journal.pone.0175566)
Supplement: S4 Fig — (A, B) Female; (C, D) Male; (A, C) Lateral view; (B, D) Dorsal view; acg–acid gland, aed–aedeagus, agl–accessory glands, alg–alkaline gland, ova–ovary, spt–spermatheca, stl–stylet of ovipositor, te–testis, val–valves of ovipositor. (PDF) [file pone.0175566.s004.pdf]

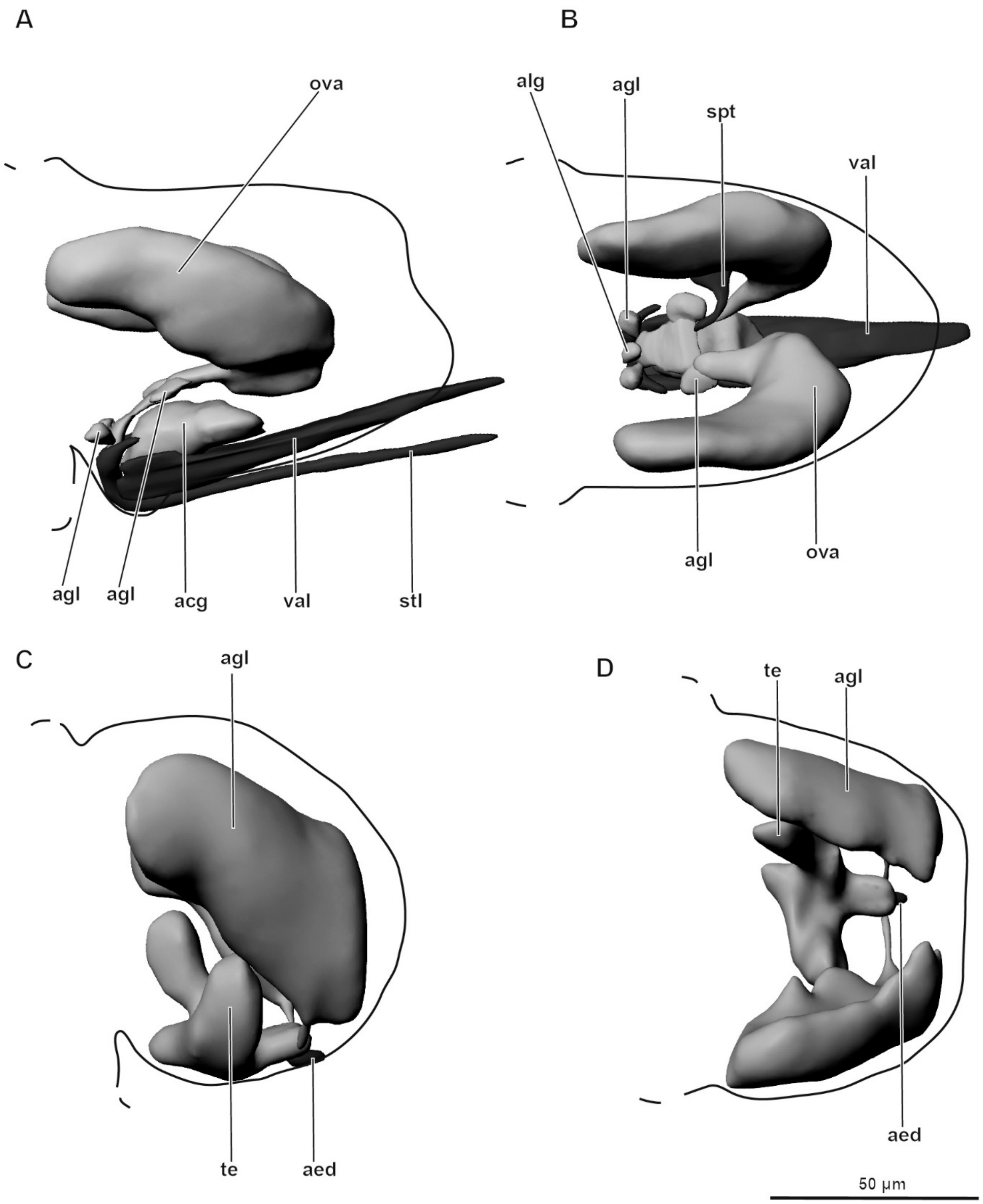

S4 Fig. Reproductive system of *Megaphragma mymaripenne*, 3D.

(A, B) Female; (C, D) Male; (A, C) Lateral view; (B, D) Dorsal view; acg – acid gland, aed – aedeagus, agl – accessory glands, alg – alkaline gland, ova – ovary, spt – spermatheca, stl – stylet of ovipositor, te – testis, val – valves of ovipositor.
