# Supplementary material for: Anatomy of adult Megaphragma (Hymenoptera: Trichogrammatidae), one of the smallest insects, and new insight into insect miniaturization
Source: PLoS One. 2017 May 3;12(5):e0175566. doi: 10.1371/journal.pone.0175566 (PMC5414980; doi:10.1371/journal.pone.0175566)
Supplement: S1 Table — (PDF) [file pone.0175566.s005.pdf]

S1 Table. Homology of head musculature in Hymenoptera

Wipfler et al. [S1] (used in this study)		v. Kéler [S2]	Matsuda [S3]	Snodgrass [S4]	Youssef [S5]
0an1	M. tentorioscapalis anterior	1	52	4	m. tent-scap I
0an2	M. tentorioscapalis posterior	2	54	2	m. tent-scap II
0an3	M. tentorioscapalis lateralis	3	51	3	m. tent-scap III
0an4	M. tentorioscapalis medialis	4	53	5	m. tent-scap VI
0lb1	M. frontolabralis	8	62	1	m. fron-labr
0lb2	M. frontoepipharyngalis	9	61		
0md1	M. craniomandibularis internus	11	21+24	9a,b	m. cran-intramand
0md3	M. craniomandibularis externus	12	23	8	m. cran-extramand
0md4	M. hypopharyngomandibularis	13	26		
0md5	M. tentoriomandibularis lateralis	-	25a		m. tent-mand
0md6	M. tentoriomandibularis lateralis	14a	25b		m. tent-mand
0md7	M. tentoriomandibularis medialis superior	-	25a		m. tent-mand
0md8	M. tentoriomandibularis medialis inferior	14b	25b		m. tent-mand
0mx1	M. craniocardinalis	15	1	10	m. geno-card
0mx3	M. tentoriocardinalis	17	3	11	m. tent-card
0mx4	M. tentoriostipitalis anterior	18	4	12, 13	m.tent-stip I, II
0la4	M. postoccipitopraementalis	-	34	17	m. postoccip-prement
0la5	M. tentoriopraementalis	29	35	18	m. tent-prement
0la6	M. tentorioparaglossalis	30	36	18	m. tent-prement
0hy1	M. frontooralis	41	74	33	m. fron-susp
0hy3	M. craniohypopharyngealis	42	37	38	m. tent-susp
0ci1	M. clypeopalatalis	43	81	25-31	m. clyp-cibpariet, m. clyp-epiph
0bu2	M. frontobuccalis anterior	45	83	34	m. fron-phar I
0bu3	M. frontobuccalis posterior	46	84	35	m. fron-phar II, ?III
0ph1	M. verticopharyngealis	51	86	36	m. cran-phar
0st1	M. annularis stomodaei	68	93	36	m. circ.phar
