# Supplementary material for: Anatomy of adult Megaphragma (Hymenoptera: Trichogrammatidae), one of the smallest insects, and new insight into insect miniaturization
Source: PLoS One. 2017 May 3;12(5):e0175566. doi: 10.1371/journal.pone.0175566 (PMC5414980; doi:10.1371/journal.pone.0175566)
Supplement: S2 Table — (PDF) [file pone.0175566.s006.pdf]

S2 Table. Homology of musculature of mesosoma in Hymenoptera

Fridrich and Beutel [S6] (used in this study)		General studies				Hymenoptera					
		Beutel abd Haas, [S7]	Snodgrass [S8]	v. Kelér [S2]	Matsuda [S9]	Duncan [S10]	Snodgrass [S4]	Alam [S11]	Gibson [S12]	Miko et all. [S13]	Vilhelmsen et al [S14]
Idlm1	M. prophragma-occipitalis	2	49	55 56	op-t 3	–	40, 41			*ph1(t1)-poc*	41
Idlm2	M. pronoto-occipitalis	1	48	57	op-t 2	–	40, 41?	43, 44		*t1-poc*	2
Idlm3	M. prophragma-cervicalis	3	56	70	cv(d)-t 1 t14	–	40, 41			*ph1(t1)-poc*	25
Idlm4	M. cervico-occipitalis dorsalis		47	–	op-t 1			43, 44			
Idlm5	M. pronoto-phragmalis anterior	6	–	71 72?	t 12			47 (I)			5
Idlm6	M. pronoto-phragmalis posterior		–	72?	t 13	l is1,2	45	46 (I)		*t1-ph1*	5
Idvm1	M. cervico-occipitalis anterior	8?	–	61	op-cv 1						15
Idvm2	M. cervico-occipitalis medialis	15	50	61	op-cv 2						15
Idvm3	M. cervico-occipitalis posterior	15	51	61	op-cv 3		42 b,c	41			15
Idvm5	M. pronoto-cervicalis anterior	14	53	60	t-cv 1						3
Idvm6	M. pronoto-cervicalis medialis	12	52	60	t-cv 2	lpm3 , 4	48	50		*t1-pl1,? ph1(t1)-pl1*	3
Idvm7	M. pronoto-cervicalis posterior	5	–	–	t-cv 3	lpm1, 2	47			*t1-cv*	4
Idvm8	M. prophragma-tentorialis	13	–	–	t-s(cv) 9		46	51			41
Idvm9	M. profurca-occipitalis	11	–	–	op-s 2 p-s 3	lfp	51	54		*fu1-cv*	26
Idvm9	M. profurca-occipitalis	11			op-s 2	Ois2	43	40+B		*fu1d-poc*	
Idvm10	M. profurca-phragmalis	16	59	87	t-s 1	58	78	63			29
Idvm16	M. pronoto-coxalis anterior	23	63	81	t-cx 5						10, 11
Idvm17	M. pronoto-coxalis posterior	23	64	81	t-cx 6 t-cx 7						10, 11
Idvm18	M. pronoto-coxalis lateralis	25	65	81	t-cx 8	Ilm6	55	57?		?*t1-cx1*	10, 11
Itpm1	M. pleurocrista-occipitalis	7	–	62	op-p 2	Ois1	42	41a+b , 42a+b		? *pl1(m, l)*-poc	
Itpm2	M. propleuro-occipitalis		57	63 64	op-p 1 t-p 3	32	42a ?	42			16, 17
Itpm3	M. pronoto-pleuralis anterior	17?	–	–	–						7
Itpm4	M. pronoto-apodemalis anterior		–	85	t-p 14?						8, 9
Itpm5	M. pronoto-apodemalis posterior		–	–	t-p 15?						8, 9

Fridrich and Beutel [S6] (used in this study)		General studies				Hymenoptera					
		Beutel abd Haas, [S7]	Snodgrass [S8]	v. Kelér [S2]	Matsuda [S9]	Duncan [S10]	Snodgrass [S4]	Alam [S11]	Gibson [S12]	Miko et all. [S13]	Vilhelmsen et al [S14]
Itpm6	M. pronoto-intersegmentalis	18	–	–	t-p 1 t-p 2	lpm5,6	49, 50	52, 53		*t1-fu1*	
Ipcm1	M. procoxa-cervicalis	26	–	–	cv-cx 3	llm2	53	55		*pl1-cx1*	
Ipcm2	M. procoxa-cervicalis transversalis		54	59	cv-cx 1 cv-cx 2	49	pl 1-cx1 cX				1
Ipcm3	M. propleuro-trochantinalis		–	86	p-ti(cx) 1						37
Ipcm4	M. propleuro-coxalis superior	24	68	82	p-cx 4						20, 32
Ipcm5	M. propleuro-coxalis inferior		68?	83	p-cx 5	llm5	57	59		*fu1-cx1l*	20, 32
Ipcm8	M. propleuro-trochanteralis	31	71 b, c	80?	p-tr 1 p-tr 2	llm3	61	61		*pl1-tr1*	21, 22
Ivlm1	M. profurca-cervicalis	10	66	0vl m3	cv-s 1 cv-s 4?	lfp	51	54		? *fu1-cv*	28
Ivlm3	M. profurca-tentorialis	9	55	58	s 1 s 2	Ois3,4	44	45		*fu1v-poc*	27
Ivlm4	M. profurca-spinalis	21	61	75	s 14 s 16						34
Ivlm7	M. profurca-mesofurcalis	42	60	74	s 13	lis4	52	48	1	*fu2-fu1v*	24, 35, 36
Ivlm9	M. prospina-mesofurcalis	43	87	77	s 12						40
Iscm1	M. profurca-coxalis anterior	27	–	89	s-cx 5	Ilm1	54	56		*s1-cx1*	105
Iscm2	M. profurca-coxalis posterior	30	66	91	s-cx 3	l lm4	56	58		*fu1-cx1m*	30
Iscm3	M. profurca-coxalis medialis		69	90	s-cx 6						31
Iscm4	M. profurca-coxalis lateralis		–	–	s-cx 2						23
Iscm5	M. prospina-coxalis	28	67	93	s-cx 4	lis3	58	60		*s2-cx1*	39
Iscm6	M. profurca-trochanteralis		–	92	s-tr1						33
IIdlm1	M. prophragma-mesophragmalis	39	81	95	t 14	IIdl1	71	62	2	*ph1-ph2*	42
IIdlm2	M. mesonoto-phragmalis	40, 41	82	96	t 12			70			49
IIdvm1	M. mesonoto-sternalis	44	83 84	100 101	t-p 5 t-p 6	IIdv1	72	111	3	*pl2-t2a*	56
IIdvm3	M. mesonoto-trochantinalis posterior		89	–	t-ti 3						53
IIdvm4	M. mesonoto-coxalis anterior	60?	90	–	t-cx 5						50
IIdvm5	M. mesonoto-coxalis posterior	60	91	105	t-cx 6 t-cx 7						50
IIdvm6	M. mesocoxa-subalaris	64	99	122	t-cx 8	–	82	72		*cx2-sa2*	67

Fridrich and Beutel [S6] (used in this study)		General studies				Hymenoptera					
		Beutel abd Haas, [S7]	Snodgrass [S8]	v. Kelér [S2]	Matsuda [S9]	Duncan [S10]	Snodgrass [S4]	Alam [S11]	Gibson [S12]	Miko et all. [S13]	Vilhelmsen et al [S14]
IIdvm7	M. mesonoto-trochanteralis	69	103	104	t-tr 1				17		51, 52
IIdvm8	M. mesofurca-phragmalis	45	–	125	t-s 1	IIdv2			11	*fu2-ph2*	75, 76
IItpm1	M. prophragma-mesanepisternalis		58?	106	t-p 3						43
IItpm2	M. mesopleura-praealaris	47	–	106	t-p 4 t-p 20	–	74		5	*pl2-t2c*	58
IItpm3	M. mesonoto-basalaris	48	–	–	t-p 7, t-p 8, t-p 9						48
IItpm4	M. mesonoto-pleuralis anterior	49	–	108	t-p 10 t-p 11 t-p 18						57
IItpm5	M. mesonoto-pleuralis medialis		–	–	t-p 12	IIpm4	75	68?	4	*pl2-t2b*	57
IItpm6	M. mesonoto-pleuralis posterior	46	–	109	t-p 15			68 (II)			57, 67
IItpm7	M. mesanepisterno-axillaris	53	–	–	t-p 13	m3Ax?	76a ?		7?	*t1-3ax2*	14
IItpm7	M. mesanepisterno-axillaris	53			t-p 13	I IIpm2a?			19	*pl2-3ax3*	14
IItpm7	M. mesanepisterno-axillaris	53				IIpm2	76b		8	*pl2-3ax2a*	14
IItpm7	M. mesanepisterno-axillaris										63
IItpm9	M. mesepimero-axillaris tertius	54	85	112	t-p 14	IIpm3	76c		9	*pl2-3ax2p*	14, 64
IItpm10	M. mesepimero-subalaris	52	–	123	t-p 16				16		
IItpm11	M. mesopleura-subalaris		–	–	t-p 19						66
IIppm1	M. mesotransanapleuralis	56	–	118	p 1						68
IIppm2	M. mesobasalare-intersegmentalis		–	–	p 2	–	–	65 (II)		*t1-ba2*	13, 38, 59
IIspm1	M. mesopleura-sternalis	50	97	115	p 3	IIpm1	77	64	10	*pl2-ba2*	62
IIspm2	M. mesofurca-pleuralis	55	86	124	p-s 1	IIfpl1	79	69	12	*pl2-fu2*	69
IIspm4	M. mesospina-intersegmentalis		–	–	p-s 6						70
IIpcm2	M. mesobasalare-trochantinalis	68	98	116	p-ti(cx) 2 p-ti(cx) 3						60
IIpcm3	M. mesanepisterno-coxalis anterior	62	96	117	p-cx 4 p-cx 6						60
IIpcm4	M. mesanepisterno-coxalis posterior	61	–	120	p-cx 5	IIlm1	80	73	13	*pl2-cx2*	71
IIpcm5	M. mesanepisterno-trochanteralis	71	103 ?	121	p-tr 2						61

Fridrich and Beutel [S6] (used in this study)		General studies				Hymenoptera					
		Beutel abd Haas, [S7]	Snodgrass [S8]	v. Kelér [S2]	Matsuda [S9]	Duncan [S10]	Snodgrass [S4]	Alam [S11]	Gibson [S12]	Miko et all. [S13]	Vilhelmsen et al [S14]
IIvlm3	M. mesofurca-metafurcalis	82	116	98	s 13	IIis2	–	71		*fu3-fu2*	81, 82
IIvlm5	M. mesospina-metafurcalis	83	117	99	s 12						83
IIscm1	M. mesofurca-coxalis anterior	65, 63?	92	126	s-cx 5	IIlm2	81	74	14	*s2-cx2*	
IIscm2	M. mesofurca-coxalis posterior	67	101	128	s-cx 3	IIlm4	83	75	15	*fu2-cx2*	77
IIscm3	M. mesofurca-coxalis medialis		100	127	s-cx 6		?83	?75 (II)			74
IIscm6	M. mesofurca-trochanteralis	72	103	129	s-tr1	67	86	76			78, 79
IIIdlm1	M. mesophragma-metaphragmalis	79	112	131	t 14	IIIdl	96		18	*ph3-ph2*	44, 45
IIIdlm2	M. metanoto-phragmalis	80, 81	112 ?	132	t 12 t 13	IIis1	70	70	42 (III)	*t2-t3*	89
IIIdvm1	M. metanoto-sternalis	84	113	135 136	t-p 5 t-p 6	IIIpm4	97–99	85-86		? *pl3-t3 (a, b)*	73, 86
IIIdvm2	M. metanoto-trochantinalis anterior	100	–	138	t-ti 1 t-ti 2						92
IIIdvm3	M. metanoto-trochantinalis posterior		118	–	t-ti 3						93
IIIdvm4	M. metanoto-coxalis anterior	101	119	140 ?	t-cx 5						91
IIIdvm5	M. metanoto-coxalis posterior	102	120	140	t-cx 6 t-cx 7						91
IIIdvm6	M. metacoxa-subalaris	105	129	153	t-cx 8	IIIp m5	105	81		*cx3-sa3*	101
IIIdvm7	M. metanoto-trochanteralis	111	133 c	139	t-tr 1	–	–			*t3-tr3*	92
IIIdvm8	M. metafurca-phragmalis	85, 86	–	156	t-s 1						110
IIItpm2	M. metapleura-praealaris	90	–	143	t-p 4 t-p 20						47
IIItpm3	M. metanoto-basalaris	93	–	–	t-p 7 t-p 8						85
IIItpm4	M. mesonoto-pleuralis anterior		–	144	t-p 10 t-p 11 t-p 18						87, 88
IIItpm5	M. metanoto-pleuralis medialis	91	–	–	t-p 12	79		? 84-86			87, 88
IIItpm6	M. metanoto-pleuralis posterior	92	–	–	t-p 15						87, 88
IIItpm7	M. metanepisterno-axillaris	96	–	–	t-p 13	IIIpm2b	100	88		*pl3-3ax3*	72, 98
IIItpm9	M. metepimero-axillaris tertius	95	114	146	t-p 14	IIIpm2b	100	89		*pl3-3ax3*	99
IIItpm10	M. metepimero-subalaris	94	–	154	t-p 16	IIIpm3a & b	102			*pl3-sa3*	

Fridrich and Beutel [S6] (used in this study)		General studies				Hymenoptera					
		Beutel abd Haas, [S7]	Snodgrass [S8]	v. Kelér [S2]	Matsuda [S9]	Duncan [S10]	Snodgrass [S4]	Alam [S11]	Gibson [S12]	Miko et all. [S13]	Vilhelmsen et al [S14]
IIItpm11	M. metapleura-subalaris		–	–	t-p 19	IIIpm3a & b	102	82		*pl3-sa3*	100
IIIppm1	M. metatransanapleuralis	97	–	–	p 1						95, 102
IIppm2	M. metabasalare-intersegmentalis		–	–	p 2						94, 96
IIIspm1	M. metapleura-sternalis	98	–	–	p 3	IIIpm1	101	87		*pl3-ba3*	94, 96
IIIspm2	M. metafurca-pleuralis	99	115	165	p-s 1						103
IIIpcm2	M. metabasalare-trochantinalis	110	127	148	p-ti(cx) 2 p-ti(cx) 3						97
IIIpcm3	M. metanepisterno-coxalis anterior	104	126	151	p-cx 4 p-cx 6			77a (III)			
IIIpcm4	M. metanepisterno-coxalis posterior	103	–	150	p-cx 5	IIIlm4	103	77a+b		? *pl3-cx3l*	106
IIIpcm6	M. metapleura-trochanteralis		–	–	p-tr 1						113
IIIvlm2	M. metafurca-abdominosternalis	fam	–	134	s 20						115
IIIscm1	M. metafurca-coxalis anterior	107	121	157	s-cx 5	IIIl m1	104	78		*pl3-cx3m*	105
IIIscm2	M. metafurca-coxalis posterior	109	123 124	159	s-cx 3	IIIlm2	106	79		*fu3-cx3(m,l)*	111, 112
IIIscm3	M. metafurca-coxalis medialis		130	159 ?	s-cx 6		?106				
IIIscm4	M. metafurca-coxalis lateralis	108	122	159	s-cx 2			79 (III)			104
IIIscm5	M. metaspina-coxalis		–	–	s-cx 4			80 (III)			
IIIscm6	M. metafurca-trochanteralis	113	133 d	160	s-tr1	IIIl m3	109	83		*pl3-tr3* ? *fu3-tr3*	107
