# Supplementary material for: Anatomy of adult Megaphragma (Hymenoptera: Trichogrammatidae), one of the smallest insects, and new insight into insect miniaturization
Source: PLoS One. 2017 May 3;12(5):e0175566. doi: 10.1371/journal.pone.0175566 (PMC5414980; doi:10.1371/journal.pone.0175566)
Supplement: S3 Table — (PDF) [file pone.0175566.s007.pdf]

S3 Table. Musculature of adult Chalcidoidea and Ichneumonoidea.

Head muscles

	0an1	0an2	0an3	0an4	0lb2	0md1	0md3	0md4	0mx1	0mx3	0mx4	0la5	0la6	0hy1	0hy3	0ci1	0bu2	0bu3	0ph1	0st1
Megaphragma	+	+	+	+	–	+	+	+	+	+	+	+	+	+	+	+	+	+	–	+
Trichogramma[1]	+	+	+	+	–	+	+	+	+	+	+	+	+	+	+	+	+	+	+	+
Anaphes[2]	+	+	+	+	+	+	+	+	+	+	+	+	+	+	+	+	+	+	+	+
Hemiptarsenus[1]	+	+	+	+	+	+	+	+	+	+	+	+	+	+	+	+	+	+	+	+

Mesosoma muscles

	Idlm1	Idlm2	Idlm5	Idvm2	Idvm5	Idvm6	Idvm7	Idvm9	Idvm18	Itpm2	Itpm3	Itpm4–	Ipcm2	Ipcm3	Ipcm4	Ipcm8	Ivlm1	Ivlm3	Ivlm7	Iscm1	Iscm2	Iscm3	Iscm5	Iscm6
Megaphragma	–	–	+	+	**a**	**a**	+	+	+	+	+	+	–	+	+	+	+	+	+	+	+	+	+	–
Trichogramma[1]	–	–	+	+	a	a	+	+	+	+	+	+	–	+	+	+	+	+	+	+	+	+	+	–
Anaphes[2]	–	–	+	+	a	a	+	+	+	+	+	+	–	+	+	+	+	+	+	+	+	+	+	–
Gonatocerus[3]	?	?	+	+	a	a	+	+	+	+	+	+	+	+	+	–	+	+	+	?	+	+	+	–
Hemiptarsenus[2]	–	+	+	+	a	a	+	+	+	+	+	+	+	+	+	+	+	+	+	+	+	+	+	–
Megastigmus[3]	?	?	+	+	a	a	+	+	+	+	+	+	+	+	+	+	+	+	+	?	+	+	+	–
Spalangia[3]	?	?	+	+	a	a	+	+	+	+	+	+	+	+	+	+	+	+	+	?	+	+	+	–
Doryctes[3]	+	?	?	+	a	a	+	+	+	–	+	+	+	+	+	+	+	+	–	?	+	+	+	+
Urosigalphus[3]	+	?	?	+	a	a	+	+	+	+	+	+	+	+	+	+	+	+	–	?	+	+	+	+

	IIdlm1	IIdvm1	IIdvm6	IIdvm7	IIdvm8	IItpm2	IItpm4	IItpm6	IItpm7	IItpm9	IItpm10	IItpm11	IIppm1	IIppm2	IIspm1	IIspm2	IIpcm4	IIvlm3	IIscm1	IIscm2	IIscm3	IIscm6	V54	V55
Megaphragma	+	–	–	+	+	+	**b**	**b**	–	+	–	–	–	+	–	+	+	**+?**	+	+	+	–	–	–
Trichogramma[1]	+	–	–	+	+	+	b	b	–	+	+	+	–	+	+	+	+	+	+	+	+	–	–	–
Anaphes[2]	+	+	–	+	+	+	b	b	–	+	+	+	–	+	+	+	+	+	+	+	+	–	–	–
Gonatocerus[3]	+	+	–	+	+	+	b	b	+	+	?	+	–	+	+	+	+	+	?	+	+	–	–	+
Hemiptarsenus[2]	+	+	–	+	+	+	+	+	–	+	+	+	–	+	+	+	+	+	+	+	+	–	–	–
Megastigmus[3]	+	+	–	+	+	+	b	b	+	+	?	+	–	+	+	+	+	+	?	+	+	–	–	+
Spalangia[3]	+	+	+	+	+	+	b	b	+	+	?	+	–	+	+	+	+	+	?	+	+	–	–	+
Doryctes[3]	+	+	+	–	+	?	b	b	+	+	?	+	+	+	+	+	+	+	?	+	+	+	–	+
Urosigalphus[3]	+	+	+	–	+	?	b	b	+	+	?	+	+	+	+	+	+	+	?	+	+	+	+	+

	IIIdlm1	IIIdvm2	IIIdvm6	IIItpm3	IIItpm5	IIItpm6	IIItpm7	IIItpm9	IIItpm11	IIIppm1	IIIspm1	IIIpcm3	IIIpcm4	IIIpcm6	IIIvlm2	IIIscm1	IIIscm2	IIIscm5	IIIscm6	V118	mx
Megaphragma	+	+	–	–	**c**	**c**	+	+	+	–	+	+	+	+	–	+	–	–	–	+	+
Trichogramma[1]	+	+	–	–	c	c	+	+	+	–	+	+	+	+	+	+	+	+	+	+	–
Anaphes[2]	+	+	–	–	c	c	+	+	+	–	+	+	+	+	+	+	+	+	+	+	–
Gonatocerus[3]	+	+	–	–	+	+	+	+	+	–	+	?	+	+	+	+	+	?	+	+	–
Hemiptarsenus[2]	+	+	–	–	+	+	+	+	+	–	+	+	+	+	+	+	+	+	+	+	–
Megastigmus[3]	+	+	–	–	+	+	+	+	+	–	+	?	+	+	+	+	+	?	+	+	–
Spalangia[3]	+	+	–	–	+	+	+	+	+	–	+	?	+	+	+	+	+	?	+	+	–
Doryctes[3]	+	–	+	+	+	+	+	+	+	+	+	?	+	+	+	+	+	?	+	+	–
Urosigalphus[3]	+	–	+	+	+	+	+	+	+	+	+	?	+	+	+	+	+	?	+	+	–

+, present; –, absent; a, b, fused muscles, ?, unknown.

[1] [29]; [2] [10]; [3] [24]
