# Supplementary material for: Anatomy of adult Megaphragma (Hymenoptera: Trichogrammatidae), one of the smallest insects, and new insight into insect miniaturization
Source: PLoS One. 2017 May 3;12(5):e0175566. doi: 10.1371/journal.pone.0175566 (PMC5414980; doi:10.1371/journal.pone.0175566)
Supplement: S1 File — (PDF) [file pone.0175566.s008.pdf]

Supplementary references

S1. Wipfler B, Machida R, Uller M, Beutel RG. On the head morphology of Grylloblattodea (Insecta) and the systematic position of the order, with a new nomenclature for the head muscles of Dicondylia. Syst Entomol. 2011; 36: 241–266.

S2. v. Kéler SV. Entomologisches Wörterbuch. Berlin: Akademieverlag; 1963.

S3.Matsuda R. Morphology and evolution of the insect head. Mem Am Entomol Ins. 1965; 4: 1–334.

S4. Snodgrass RE. The skeleto-muscular mechanisms of the honey bee. Smithson Misc Collect. 1942; 103: 1–120.

S5. Youssef N. Topography of the cephalic musculature and nervous system of the honey bee *Apis mellifera* Linnaeus. Smithson Contrib Zool. 1971; 99: 1–54.

S6. Friedrich F, Beutel RG. The thorax of *Zorotypus* (Hexapoda, Zoraptera) and a new nomenclature for the musculature of Neoptera. Arthropod Str Devel. 2008; 37(1): 29–54.

S7. Beutel RG, Haas A. Phylogenetic releationships of the suborders of Coleoptera (Insecta). Cladistics. 2000; 16: 103–141.

S8. Snodgrass RE The thoracic mechamism of a grasshopper, and its antecedents. Smithson Misc Collect. 1929; 82: 1–112.

S9. Matsuda R. Morphology and evolution of the insect thorax. Mem Entomol Soc Can Suppl. 1970; 76: 1–431.

S10. Duncan CD. A contribution to the biology of North American vespine wasps. Stanford Univ Pub Biol Sci. 1939; 8: 1–272.

S11. Alam SM. The skeleto-muscular mechanism of *Stenobracon deesae* Cameron (Braconidae, Hymenoptera) - An ectoparasite of sugarcane and juar borers of India Part I Head and thorax. Aligarh Muslim Univ Pub Ind Insect Types. 1951; 3: 1–174.

S12. Gibson GAP. Evidence for monophyly and relationships of Chalcidoidea, Mymaridae, and Mymarommatidae (Hymenoptera: Terebrantes). Can Entomol. 1986; 118: 205–240.

S13. Mikó I, Vilhelmsen L, Johnson NF, Masner L, Penzes Z. Skeletomusculature of Scelionidae (Hymenoptera:Platygastroidea): head and mesosoma. Zootaxa. 2007; 1571: 1–78.

S14. Vilhelmsen L, Miko I, Krogmann L. Beyond the wasp-waist: structural diversity and phylogenetic significance of the mesosoma in apocritan wasps (Insecta: Hymenoptera). Zool J Linn Soc. 2010; 159(1): 22–194.
